# Multimodal fNIRS–EEG sensor fusion: Review of data-driven methods and perspective for naturalistic brain imaging

**DOI:** 10.1162/IMAG.a.974

**Published:** 2025-11-05

**Authors:** Tomás Codina, Benjamin Blankertz, Alexander von Lühmann

**Affiliations:** BIFOLD—Berlin Institute for the Foundations of Learning and Data, Berlin, Germany; Intelligent Biomedical Sensing (IBS) Lab, Technical University of Berlin, Berlin, Germany; Neurotechnology Group, Technische Universität Berlin, Berlin, Germany

**Keywords:** functional near-infrared spectroscopy (fNIRS), diffuse optical tomography (DOT), electroencephalography (EEG), multimodal fusion, source decomposition

## Abstract

Functional near-infrared spectroscopy (fNIRS), high-density diffuse optical tomography (HD-DOT), and electroencephalography (EEG) are established, cost-effective, and non-invasive neuroimaging techniques, whose integration represents a promising direction for brain activity decoding with high spatiotemporal resolution in naturalistic scenarios. However, robust machine-learning methods for combining these signals remain challenging. In this review, we focus on multimodal fusion methods, emphasizing data-driven unsupervised symmetric techniques, and study their performance on our own HD-fNIRS–EEG data with synthetic ground truth. To this end, we performed a systematic method-oriented survey on fNIRS/DOT–EEG fusion, categorizing works based on fusion strategies, and identifying common artifact removal techniques and integrated auxiliary signals. Our review indicates that while many studies incorporate robust artifact handling for EEG, confounder correction in fNIRS remains limited to filtering or motion removal. Moreover, short-separation measurements and other auxiliary signals for fNIRS remain underutilized. Fusion methods predominantly rely on data concatenation, model-based, or decision-level strategies, while source-decomposition techniques are underrepresented, despite their potential for revealing more complex latent neurovascular coupling processes. To address the scarcity of multimodal public datasets, we generated a realistic synthetic HD-fNIRS–EEG dataset that simulates a finger tapping motor task, with concurrent suppression of EEG alpha-band power and an increase in hemoglobin in fNIRS from a shared neuronal source. We illustrate a proof-of-concept comparison of some source-decomposition methods on this dataset and provide the full implementations and an example Jupyter notebook to reproduce and extend these results.

## 1 Introduction: Motivation and Scope

Human neuroscience and neurotechnology are on the verge of a significant transformation, moving from conventional laboratory settings toward embracing the complexity of natural environments (Park et al., 2018; Pinti et al., 2020; Schmuckler, 2001; Sonkusare et al., 2019; von Lühmann et al., 2021). This shift toward an ecologically valid depiction of human brain function promises to provide new scientific insights and breakthroughs in our understanding of neuronal development, health, and aging, and drive translation in medicine and psychiatry. However, substantial interdisciplinary challenges need to be overcome as these settings elude many of the conventional methods, sensors, and parametric experimental paradigms currently available. To better understand the complex interactions between brain networks, the body, and the environment, it is paramount to develop integrative multimodal platforms that can continuously monitor the embodied brain (Kiverstein & Miller, 2015; Scholkmann et al., 2022) and discover and model the intertwined complex interactions between physiology, behavior, and cognition in everyday settings.

### 1.1 EEG and fNIRS

Wearable functional near-infrared spectroscopy (fNIRS), high-density diffuse optical tomography (HD-DOT), and electroencephalography (EEG) are technologies that offer such a pathway. fNIRS and EEG are established, safe, and cost-effective non-invasive neuroimaging techniques that allow brain imaging under naturalistic settings. In recent years, wearable fiberless continuous wave (CW) fNIRS systems and wearable EEG have made rapid progress (Ayaz et al., 2012; Casson et al., 2010; Niso et al., 2023; Pinti et al., 2018; Vidal-Rosas et al., 2023; von Lühmann et al., 2021; Zhao & Cooper, 2017). EEG and fNIRS complement each other by combining EEG’s ability to capture synchronous neuro-electrical activity with millisecond time scale and fNIRS’ ability to measure slow changes in cerebral blood flow with better spatial localization. Both measures, while sensitive to different types of physiology and measurement artifacts, are linked via neurovascular coupling (NVC) (T. Huppert et al., 2017; Keles et al., 2016; Logothetis, 2008), which relates transient neural activity to subsequent hemodynamic changes, the latter corresponding to BOLD signals in fMRI. Consequently, multimodal neuroimaging with EEG–fNIRS is highly promising for many research disciplines such as brain–computer interfaces (BCI) (Fazli et al., 2012; Hong et al., 2018; von Lühmann, 2018), clinical neurology (Abtahi et al., 2020; Schneider et al., 2015; Sirpal et al., 2019; Wallois et al., 2010) and neurorehabilitation (Berger et al., 2019; Mihara & Miyai, 2016). Wearable EEG and fNIRS instruments and first combined fNIRS–EEG devices are increasingly available (Safaie et al., 2013; Uchitel et al., 2021; von Lühmann et al., 2017). Furthermore, to realize the full potential of fNIRS, high-density (HD) arrays of multiple source–detector separations (commonly less than 15 mm to 4 cm) with overlapping sensitivity distributions can be employed to enable three-dimensional image reconstruction of functional activation (Arridge & Schotland, 2009; Bluestone et al., 2001; Boas et al., 2001; Wheelock et al., 2019; Zeff et al., 2007), referred to as high-density diffuse optical tomography (HD-DOT). Recent HD-DOT studies have achieved the same spatial resolution of the entire surface of the brain as the latest fMRI-based Human Connectome Parcellation scheme (Habermehl et al., 2012; White & Culver, 2010), enabling connectivity analysis or decoding performance comparable with fMRI (Eggebrecht et al., 2014; Ferradal et al., 2016; Tang et al., 2023). Complemented by recent advances in inferring subcortical from cortical network activity (Pang et al., 2023), fNIRS-based HD-DOT is now a viable extension of fMRI (Anaya et al., 2023; Vidal-Rosas et al., 2023) for the everyday world, which can offer the opportunity for seamless integration with EEG.

### 1.2 Artifacts

Despite their great potential upon fusion, it is a highly non-trivial challenge to distinguish evoked neuronal from complex systemic physiological activity in fNIRS (T. J. Huppert, 2016; Scholkmann et al., 2022; Yücel et al., 2015), and EEG (Parra et al., 2005; Urigüen & Garcia-Zapirain, 2015) already in well-controlled conventional experiments, a problem that is greatly amplified by natural behavior and motion. fNIRS and EEG are contaminated by hemodynamic and bioelectric signals from systemic, non-neural (extracerebral) physiology, which can mask the underlying neural activation and reduce contrast. While many of these confounder signals, such as cardiac, respiratory, muscle, and blood pressure changes, affect both modalities, they manifest with distinct temporal, spatial, and amplitude characteristics in each modality. More precisely, while indeed the same physiological source can originate artifacts in both modalities, it leads to a very different vascular and bio-electrical manifestation (like cardiac activity leading to electrical ECG spikes and hemodynamic pulse waves). Given their different physical measurement principles, both modalities are “blind” to each other’s case, making their combined analysis complementary. In EEG, contamination is primarily caused by ocular activity (EOG) and head and neck muscle activity (EMG), which increase variance across much of the relevant EEG spectrum. Neuronal and non-neural field potentials (e.g., EOG) traverse the tissue quasi-instantaneously. This is not true for fNIRS. In fNIRS, the contamination comes from the scalp, brain motion in the cerebro-spinal fluid (CSF), and the brain vasculature (T. J. Huppert, 2016; Scholkmann et al., 2022; Yücel et al., 2015, 2021). Hemodynamics and blood flow are affected by physical activity, induced and spontaneous blood pressure changes, breathing, heart rate and partial pressure of CO_2_, and can have a major impact on task-evoked and resting-state fNIRS signals (Franceschini et al., 2006; Funane et al., 2014; Goodwin et al., 2014; Kirilina et al., 2012; Mesquita et al., 2010). Due to the physiological complexity of hemodynamics in vasculature, channels measure a unique mixture of global and local physiological and neuronal activity, both evoked by and independent from stimuli and behavior. In contrast to EEG, these different hemodynamic signal components are non-instantaneously coupled into measurement channels with spatially and behaviorally (e.g., motion) dependent non-stationary delays and morphology as they disperse along the arteriole system and undergo vasomodulation. The shared latent neurovascular link between fNIRS and EEG, the fact that their instruments can be wearable, and the complementary nature of their physiology, artifacts, and noise make EEG and fNIRS promising candidates for fusion-based multimodal neuroimaging in general, and in the everyday world in particular.

### 1.3 Data-driven approaches

While model/regression-based methods such as the General Linear Model (GLM) in fNIRS offer well-established approaches for the analysis of conventional parametric fNIRS and EEG experiments, they usually fall short when (1) precise knowledge about stimuli and timing is not available or (2) latent complex physiological relationships and coupling mechanisms are unknown. Data-driven approaches based on machine learning can help alleviate this problem (von Lühmann, Li, Müller, et al., 2020; von Lühmann et al., 2019) and have seen a steep rise in the last decade (Zhuang et al., 2020). However, there is still a significant lack of established data-driven methods that combine fNIRS/DOT and EEG to harness *unsupervised symmetric (bidirectional) multimodal sensor fusion* to robustly model, identify, and extract co-modulation or reject physiological confounders. As our review of the existing literature on EEG–fNIRS fusion will reveal, most existing methods either require supervision through a priori labeling or modeling or do not fuse information bidirectionally on the sensor level, where physiological interactions and confounders can best be taken into consideration. We consider that this lack of well-established methods for unsupervised fusion, which is independent of labels or stimulus timing, is particularly problematic for (1) the analysis of data from continuous brain imaging in naturalistic environments, (2) the assessment of (dynamic) brain network activity in functional connectivity analysis, and (3) more advanced preprocessing in hybrid EEG–fNIRS single trial analysis, for example, for BCI. This set of problems fully motivates the focus of this review.

### 1.4 Related reviews

Due to its great promise, multimodal EEG–fNIRS integration has been reviewed already in other occasions from different perspectives. We identified 10 methods-oriented review articles, all except one focusing on specific domains of application. In the context of hybrid BCI, Ahn and Jun (2017) surveyed studies implementing EEG–fNIRS fusion methods for general BCI purposes, J. Chen et al. (2023) reviewed previous works for motor rehabilitation, Hong et al. (2018) covered feature extraction and classification algorithms for locked-in syndrome, H. Khan et al. (2021) analyzed articles focusing on human gait, and AlQahtani et al. (2024) focused on lower prosthetic limbs for rehabilitation. Other method-oriented reviews surveyed articles utilizing EEG–fNIRS fusion methods in the context of robot-assisted gait rehabilitation (Berger et al., 2019), diverse subdomains of neuroergonomics (Bourguignon et al., 2022), neurovascular coupling analysis (Yeung & Chu, 2022), and high-frequency cerebral physiology analysis (Vakitbilir et al., 2024). A general-purpose methodology-focus review was presented in R. Li et al. (2022), where the authors conducted an exhaustive literature search ending up with 92 selected studies. The latter were separated into three categories depending on the fusion strategy: EEG-informed fNIRS analyses, fNIRS-informed EEG analyses, and parallel fNIRS–EEG analyses. These studies, covered in R. Li et al. (2022)’s work, alongside their categorization, were also considered in our survey.

### 1.5 Motivation and scope

While the reviews mentioned above provide a thorough and valuable overview of many EEG–fNIRS fusion approaches, a particular domain of methods has barely received attention (see categorization in the next section and Fig. 2): Unsupervised data-driven methods for parallel/symmetric fNIRS–EEG fusion. *We hypothesize that these methods are particularly valuable for the analysis and decoding of data from continuous brain imaging in naturalistic everyday-world environments, and in the presence of physiological artifacts*. To fill this gap, we focus our work on this domain, updating and complementing previous reviews with a perspective on source-decomposition multimodal fusion methods. Furthermore, due to the community’s general interest in physiological interpretability, we restrict our study to classical machine-learning (ML) methods, as interpretation of their deep-learning (DL) counterparts is still in large parts an ongoing effort (Eastmond et al., 2022). With the sole purpose of distinguishing these methods and then excluding the DL ones, we define the latter as neural networks with two or more non-linear layers. We emphasize that such a definition plays a purely practical role, and should not be taken as a rigorous, global definition of the concept. For a comprehensive review of DL methods for fNIRS/DOT, we refer the reader to our recent article (Dissanayake et al., 2025). On top of our method-oriented survey, we also perform a qualitative and quantitative comparison of what we consider are the most promising data-driven unsupervised symmetric sensor fusion methods for multimodal source decomposition. Their performance is assessed on a realistic HD-fNIRS–EEG data with synthetic ground truth developed for this purpose. We make all compared source-decomposition methods available to the community in our Python-based fNIRS/DOT analysis toolbox Cedalion (IBS-Lab & Cedalion-Developers, 2024),^1^ and we provide a link to an example notebook^2^ to enable researchers to reproduce and explore our results based on the same synthetic data (see Supplementary Materials).

It is worth noting that our emphasis on source-decomposition methods relative to model-based, asymmetric, or non-directional approaches arises mainly from our perspective on continuous naturalistic brain imaging (von Lühmann et al., 2021) and should not be construed as a general endorsement of these techniques as the optimal machine-learning solutions for all neuroscience applications.

### 1.6 Review structure

The rest of the paper is organized as follows: In Section 2, we provide a taxonomy and categorization of fNIRS–EEG fusion methods. In Section 3, we present the results of our method-oriented survey of fusion approaches with a particular focus on data-driven unsupervised symmetric methods. In Section 4, we introduce our realistic simulated HD-fNIRS–EEG data and use it to compare the performance of some source-decomposition methods. In Section 5, we conclude the review with a discussion of current challenges, limitations, and future directions.

## 2 Taxonomy and Overview of Fusion Strategies

### 2.1 EEG–fNIRS fusion

Multimodal data offer a significant advantage over single modalities when it provides complementary information of the same underlying phenomena. Fusion methods play a crucial role in enhancing the interpretation and utility of such multimodal data by leveraging individual strengths, while mitigating the effect of modality-specific confounding signals. In particular, EEG–fNIRS fusion capitalizes on the temporal accuracy of EEG in detecting electrical signals and the spatial accuracy of fNIRS in locating these signals within specific brain regions. Additionally, due to the different nature of the acquisition principles, most artifacts are not shared between modalities. Consequently, EEG–fNIRS fusion methods are promising data analysis techniques for NVC investigation, and brain activity decoding with improved spatiotemporal and signal-to-noise ratio (SNR) when compared with their individual application.

### 2.2 Fusion-level taxonomy

**Fig. 1.**
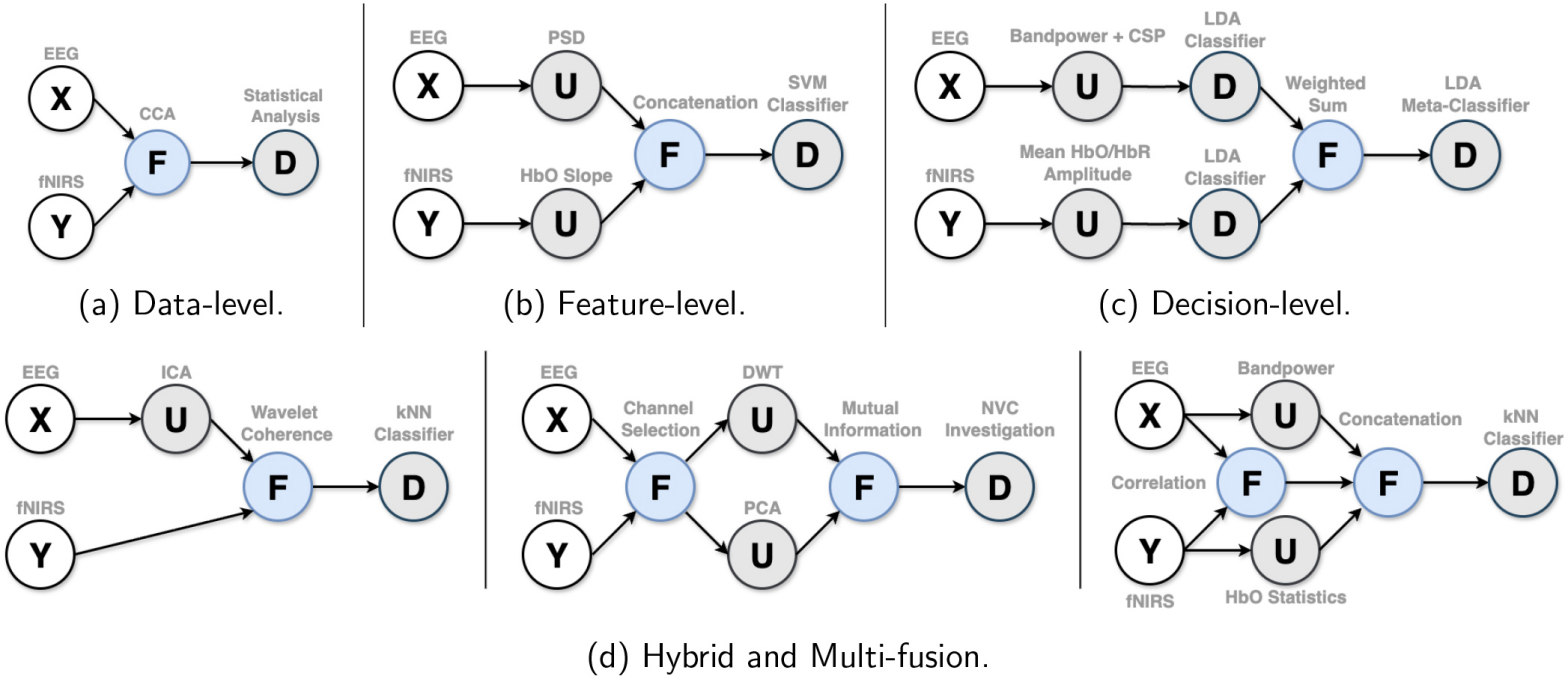
Stages or levels at which fusion methods can be integrated into the data analysis pipeline: **X** and **Y** represent EEG and fNIRS-derived multivariate time series, respectively, including raw sensor signals. The **U**, **F**, and **D** nodes represent unimodal processes, the proper fusion methods, and decision algorithms, respectively. The gray texts provide some concrete examples of these processes.

Fusion methods can be classified by their location along the data analysis pipeline, leading to the following categories: **Data-Level Fusion** (Fig. 1a) happens at the beginning of the pipeline, combining raw, or very low-processed data.^3^ They tend to capture a more detailed picture of brain activity by exploiting modality features that might be lost in later stages of processing. Typical challenges include handling the inherent differences between the nature of the datasets, synchronizing and aligning data, and dealing with high-dimensionality problems. **Feature-Level Fusion** (Fig. 1b) takes place after single-modality features are generated individually. The unimodal prior steps can alleviate high-dimensionality and data-incompatibility problems, by bringing datasets into a common (low-dimensional) feature space. However, loss of relevant information may occur at this step. In **Decision-Level Fusion** (Fig. 1c), independent decisions derived from single modalities are aggregated to produce a final output. They can be useful when each modality assesses an independent aspect of brain activity, but they can also fail to exploit complementary information due to late fusion. On top of these three cases, in practice, we often encounter hybrid strategies, where each modality enters the fusion at a different stage (Fig. 1d left), or cases in which modalities are combined at multiple stages (Fig. 1d middle and right).

### 2.3 Model-based and data-driven

**Fig. 2.**
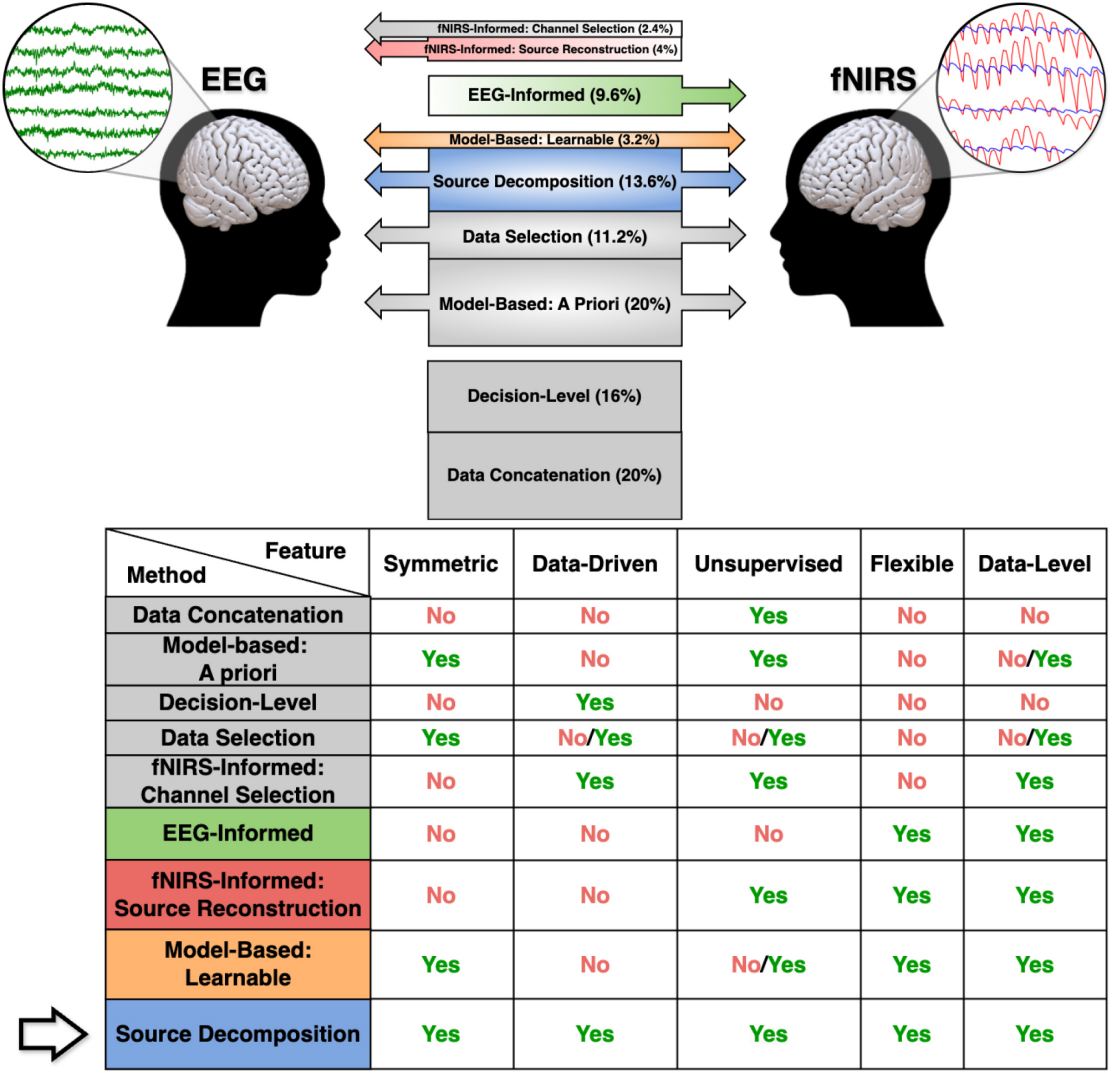
Taxonomy of EEG–fNIRS fusion strategies. Top: Organization of the nine categories into a vertical stacked bar plot, where bar thickness reflects the percentage of articles from our survey that used each fusion strategy. The right/left arrows on each box represent the directionality of the method, namely whether they are modality symmetric (left and right), EEG-informed (right), fNIRS-informed (left), or non-directional (none). Bottom: Table of category features, relevant for single-trial applications in continuous brain imaging. As indicated by the arrow, in this review, we are particularly focused on source-decomposition methods, since they contain all those desired properties. In both subfigures, colored categories represent the most promising fusion strategies for capturing more complex relationships due to their flexibility and early fusion implementations.

In terms of functionality, EEG–fNIRS fusion methods can be further distinguished between model-based versus data-driven, and asymmetrical versus symmetrical, leading to the nine categories depicted in Figure 2. Model-based algorithms utilize theoretical models and a priori knowledge to parameterize an underlying phenomenon while data-driven methods leverage data to uncover patterns, primarily through statistical techniques. When an accurate theoretical representation is used, model-based methods can outperform data-driven ones in terms of interpretability and precision, while for incomplete or inapplicable models, data-driven methods offer more flexibility and adaptability to learn complex patterns from the data (von Lühmann, Li, Müller, et al., 2020; von Lühmann et al., 2019).

### 2.4 Asymmetric, symmetric, and non-directional

Asymmetric methods make use of one of the modalities to enhance the signal quality of the other (R. Li et al., 2022). In these unilateral approaches, the former modality does not enjoy the benefits of multimodality. EEG-informed algorithms exploit EEG high temporal resolution to guide fNIRS processing, improving the quality of the reconstructed hemodynamic response (Y. Gao, Jia, et al., 2023; Peng et al., 2014; Pouliot et al., 2014). fNIRS-informed strategies, in contrast, leverage fNIRS higher spatial resolution to restrict EEG analysis to specific regions of interest in channel (channel selection) (R. Li et al., 2017; Moslehi & Davies, 2021) or volume space (source reconstruction) (Cao et al., 2021; R. Li et al., 2019). The latter typically makes use of head models for better source localization (Aihara et al., 2012), which increases computational and memory demands. From our survey, we found that most EEG-informed fusion methods were model-based and supervised (labeled data), via GLM-based fNIRS analysis, while fNIRS-informed algorithms were typically unsupervised (unlabeled data).

In EEG–fNIRS symmetric methods, both modalities inform and are informed by the other. They can be divided into model-based or data-driven, depending on the amount of prior knowledge incorporated during fusion. The former category is further split depending on whether the method contains parameters that must be learned during training (learnable) (Y. Gao, Liu, et al., 2023; Sood et al., 2016) or whether they are static, “handcrafted” functions (a priori) (Aghajani et al., 2017; Giacometti & Diamond, 2014; Othman et al., 2021). Such a priori knowledge is typically derived from human expertise rather than data, making the model less flexible and hence less capable of capturing single-trial variability. This is the case of many NVC-like features, which combine modalities via a pre-defined function, such as correlation (L.-C. Chen et al., 2015), that models the relationship. Symmetric data-driven approaches are further separated into data selection and source-decomposition methods. The former focuses on selecting (without modifying) a subset of the most informative input data (Deligani et al., 2021; Hasan et al., 2020). Source-decomposition fusion methods (Zhuang et al., 2020), also known as latent component analysis, assume each dataset is generated from a mixture of latent (unobserved) sources that must be identified by the algorithm and are assumed to co-modulate between modalities. Apart from the choice of co-modulation function, which defines the algorithm (Witten et al., 2009), these highly flexible and unsupervised methods are purely data-driven, making them ideal for capturing complex structures for which no good a priori model is known (Dähne et al., 2013; Dashtestani et al., 2022).

On top of these categories, we have data concatenation (Almajidy et al., 2015; Cao et al., 2022) and decision-level fusion (Fazli et al., 2012; Pfurtscheller, 2010; Shin, Kim, et al., 2018), corresponding to non-directional methods that take both modalities as inputs but, unlike directional approaches, they do not use the output to inform any of the modalities during processing. The former strategy concatenates features into a unified vector and then passes it as input of a decision-making algorithm, while the latter makes independent single-modality decisions and then combines them to produce a final output. Both methods are simple to implement and computationally cheap, but they may struggle to capture the underlying complex relationships between modalities.

### 2.5 Relevance for continuous brain imaging

Such a taxonomy allows us to identify what we consider are the most promising EEG–fNIRS fusion strategies for capturing more complex relationships between modalities (colored in Fig. 2) due to their flexibility and early fusion implementations. On top of these features, source-decomposition methods are also data-driven, and unsupervised (Fig. 2 bottom), making them good candidates for successful single-trial applications in continuous brain imaging data, for example, from the everyday world. Despite the potential benefits of the flexible methods (colored) over the others (gray), the former seem to be underrepresented in current studies according to our survey (Fig. 2 top), where more than half of the articles implement selection, model-based a priori, or non-directional approaches. In particular, most of the BCI-related articles utilize some of these inflexible methods.

## 3 Survey of Fusion Methods

### 3.1 Preliminaries

#### 3.1.1 Methodology

**Fig. 3.**
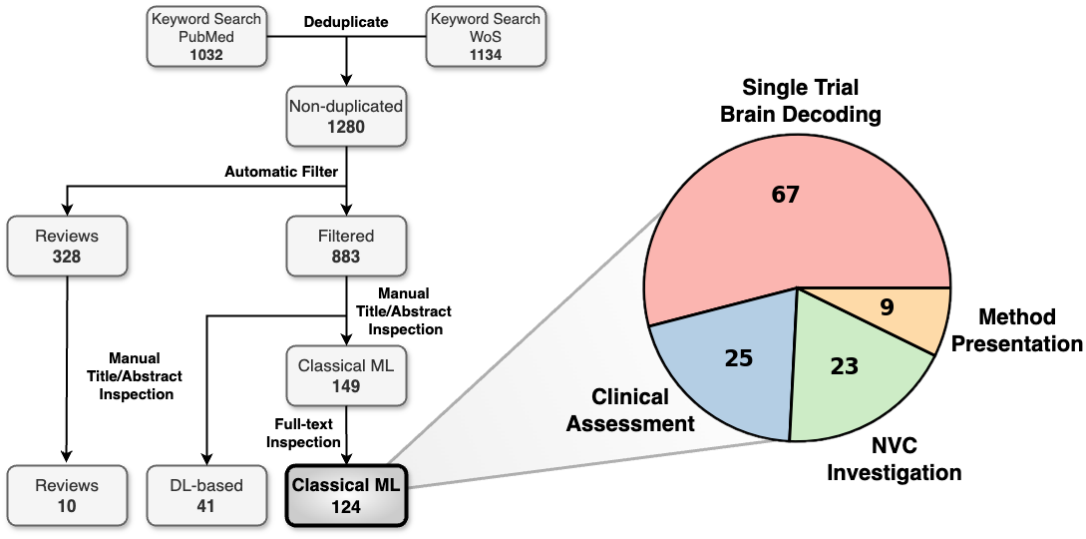
Flow diagram of the conducted survey and distribution of research areas for the selected 124 articles using classical ML-based EEG–fNIRS fusion methods.

The survey was conducted using PubMed and Web of Science (WoS) databases, comprising articles until March 25, 2025, with no lower limit on publishing date (Fig. 3). We searched for English articles containing the combination of keywords (“fNIRS” OR “NIRS” OR “functional near-infrared spectroscopy” OR “near-infrared spectroscopy”) AND (“EEG” OR “electroencephalography”) in the title OR abstract, obtaining 1032 and 1134 results for PubMed and WoS, respectively. Eliminating duplicates, we ended up with 1280 articles. As a preliminary automatic filter, we excluded preprints, comment articles, retractions, and erratum, and separated apart for later inspection 328 reviews, leading us to 883 published articles. From this subset, we initiated a manual filtering via title and abstract inspection, excluding articles that involved non-human subjects, were dataset or instrumentation oriented, or did not use fusion methods but rather unimodal analyses in parallel. This manual screening returned 190 articles, including 41 using DL-based methods. The remaining 149 documents went through a full-text reading procedure, revealing 25 additional nonrelevant articles, according to the criteria mentioned above. The final selection consisted of 124 non-duplicated, journal-published articles involving EEG–fNIRS classical ML fusion methods, to which we restrict all further analyses in this review. Publishing dates range from April 2010 (Pfurtscheller, 2010) up to March 2025 (Saricaoglu et al., 2025), most of them from Q1 and Q2 journals, such as IEEE TNSRE, IEEE TBME, IEEE ACCESS, Nature Scientific Reports, NeuroImage, Frontiers in Neuroscience, and MDPI Sensors. The 328 separated review articles were scrapped via manual title/abstract filtering ending up with 10 methodology-focus EEG–fNIRS fusion-method reviews, which were already introduced in Section 1. During this search, we found no novel EEG–fNIRS fusion methods that had not been included already from the previous 883 surveyed articles.

#### 3.1.2 DOT-EEG

As diffuse optical tomography (DOT) is a field increasingly on the rise, we also conducted a survey on DOT–EEG fusion methods in a similar fashion, using the same search engines and publishing date ranges. We searched for English articles with the keyword combination (“DOT” OR “diffuse optical tomography” OR “diffuse optical imaging”) AND (“EEG” OR “electroencephalography”) in the title OR abstract. We found 243 and 319 documents in PubMed and Wos, respectively, which, upon deduplication, reduced to 367, including 15 review articles. We then performed a manual filtering via title and abstract inspection, using the same criteria as for the fNIRS–EEG survey, ending up with 5 articles using classical ML-based DOT–EEG fusion methods, and no relevant reviews.^4^

#### 3.1.3 Research areas

The survey revealed a diverse range of applications for EEG–fNIRS fusion methods, which allowed us to group the 124 articles into 4 categories, whose distribution is shown in Figure 3. From the EEG–DOT fusion methods survey, out of the five selected articles, we found one in the area of clinical assessment, two in NVC investigation, and two in method presentation. Single trial brain decoding encompasses more than half the survey volume, containing studies that investigate brain decoding during experimental tasks such as motor, mental workload, and cognitive load, most of them focusing on hybrid BCI for brain state classification. Articles in the clinical assessment category make use of EEG–fNIRS systems for diagnosing, monitoring, and predicting various neurological and psychiatric conditions such as epilepsy, ADHD, depression, and Alzheimer’s Disease (AD). NVC investigation contains publications that exploit simultaneous EEG and fNIRS recordings to model or perform a quantitative analysis of NVC, sometimes through functional connectivity analysis (FCA). Method presentation groups all studies whose main goal and contribution are the development of a new data analysis method.

#### 3.1.4 Survey results and implications for continuous brain imaging

**Fig. 4.**
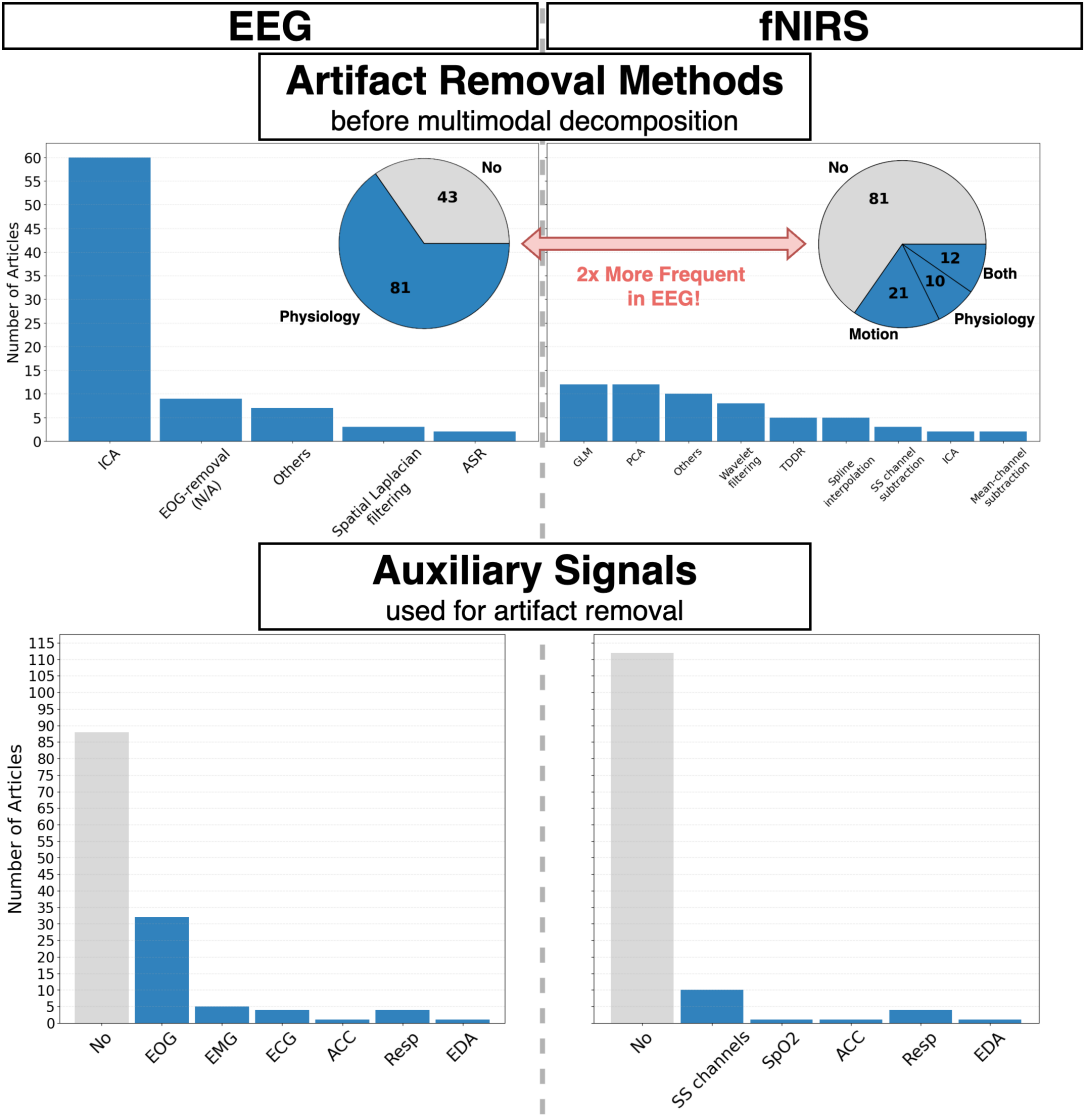
Distribution of artifact removal techniques (top) and auxiliary signals (bottom) used for EEG (left) and fNIRS (right) from surveyed studies. The category “Others” contains non-specified artifact removal methods, as well as algorithms that were found only once during the survey.

Before diving into details, we summarize our survey’s main results and their relevance for the main topic of the review, continuous brain imaging. Regarding data preprocessing (Section 3.2), the following conclusions were drawn (Fig. 4):

- Most studies using fNIRS/DOT–EEG fusion methods do not sufficiently address physiological confounders in fNIRS/DOT. Instead, they typically incorporate simple channel pruning and filtering techniques or handle motion artifacts only. In contrast, most of the studies do incorporate confounder removal techniques for EEG, such as EOG, EMG, or ECG regression.
- Most studies using fNIRS/DOT–EEG fusion methods do not incorporate auxiliary signals, and when they do, they are mostly used to track EEG-related artifacts such as EOG or EMG, but not for monitoring fNIRS confounders or artifacts. In particular, short-separated (SS) channels are rarely recorded.^5^

These findings reveal a great potential for the neuroimaging community to improve fNIRS/DOT–EEG fusion methods interpretability and reliability by comprehensively addressing physiological confounders and artifact removal in fNIRS data.

The taxonomy introduced in Section 2 allowed us to group all surveyed EEG–fNIRS/DOT fusion strategies into the nine categories of Figure 2. There, one can also see the fNIRS–EEG methods distribution (while from the DOT–EEG survey, we found two and three articles in the “Model-Based: A priori” and “fNIRS-Informed: Source Reconstruction” categories, respectively). *The reason for such a customized taxonomy comes from the need to identify what we consider are the most promising fusion methods for capturing complex relationships between modalities*, namely EEG-informed (Section 3.3), fNIRS-informed: Source Reconstruction (Section 3.4), Model-based: Learnable (Section 3.5), and Source Decomposition (Section 3.6). Articles in these categories will be described in detail in the corresponding sections, while the works using nondirectional strategies are given in Table A1 (Concatenation) and Table A2 (Decision-Level) in Appendix A. Here we summarize the survey’s results regarding fusion methods as follows (Fig. 2):

- Most of the articles utilize only data concatenation (20%), model-based: a priori (20%), or decision-level (16%) approaches. The remaining articles utilize mostly asymmetrical (16%) or selection (11.2%) algorithms.
- The domain of source-decomposition algorithms (13.6%) seems to be underrepresented. The approaches we identified (such as the CCA and jICA methods family, and mSPoC) do not address/remove physiology in fNIRS before or during fusion.

While these findings do not represent a problem for conventional neuroscience applications, we do believe further work is needed to tackle the challenging scenarios that arise from naturalistic brain imaging. From this perspective, the most utilized methods so far may not be enough to uncover complex patterns due to their non-directional nature, lack of learnable parameters, or late fusion. Moreover, while asymmetrical methods have been successfully applied to capture inter-trial variability (Section 3.3.1) or achieve higher spatiotemporal resolution than each individual modality (Section 3.4.1), within them, one of the modalities does not fully profit from fusion, or, in other words, not both modalities inform each other. We believe that such asymmetrical treatment represents a sub-optimal solution that can be cured by moving forward to bi-directional data-driven methods, such as multimodal source-decomposition methods. Consequently, we argue that the latter deserve more attention, due to their great potential to better find shared NVC latent processes, while also simultaneously rejecting physiology and motion artifacts.

In light of these findings and the review’s perspective, we ranked the source-decomposition methods identified in our survey by evaluating their performance using the same synthetic dataset. While a more comprehensive analysis will be conducted in subsequent work, the current results provide first valuable insights into each method’s specific characteristics, strengths, and limitations.

### 3.2 Artifact removal techniques and auxiliary signals

EEG and fNIRS data are both susceptible to artifacts that can compromise signal quality and contrast-to-noise ratio, with EEG being influenced by electrophysiological noise (e.g., eye blinks, muscle activity) and external electromagnetic interference, and fNIRS being affected by cardiovascular hemodynamics and ambient optical noise. Motion exacerbates artifacts in both modalities, making advanced preprocessing techniques (even prior to fusion) necessary for reliable neuroimaging in naturalistic environments. Single-modality preprocessing typically involves channel pruning, bandpass filtering (targeting brain state frequencies in EEG and hemodynamic responses in fNIRS), re-referencing (EEG), converting light intensity to hemodynamic changes by using the modified Beer-Lambert law (fNIRS), detrending, and normalization. Within such preprocessing pipelines, we are particularly interested in artifact removal techniques and the use of auxiliary physiological signals. For EEG (Islam et al., 2016), regression methods and ICA are typically used to remove EOG, EMG, and ECG interference, while fNIRS implements methods such as wavelet filtering (Chiarelli et al., 2015; Molavi & Dumont, 2012), Principal Component Analysis (PCA) (X. Zhang et al., 2016), and spline interpolation (Scholkmann et al., 2010) to reduce motion artifacts, and more sophisticated algorithms, such as the General Linear Model (GLM) (Diamond et al., 2006; Kirilina et al., 2013) and (when SS channels are available) SS-channel regression (Gagnon et al., 2011; Yücel et al., 2015), to model physiological noise. Additionally, both modalities benefit from spatial filtering, where multi-sensor signals are combined to enhance brain activity components.

Auxiliary signals are often incorporated into noise reduction algorithms to improve contrast. For EEG (Islam et al., 2016), common examples include EOG and EMG to reduce ocular and muscular artifacts, and ECG to track cardiac interference. For fNIRS (Scholkmann et al., 2022; Tachtsidis & Scholkmann, 2016), heart rate is commonly used to track systemic cardiac activity, photoplethysmography (PPG) and blood pressure to monitor vascular dynamics, and slower oscillations such as Mayer waves, respiration to account for breathing-related fluctuations, and SpO_2_ (oxygen saturation) to assess systemic oxygenation changes. Additionally, SS channels can be incorporated to capture global systemic, non-cerebral hemodynamic activity. In both modalities, accelerometers and gyroscopes help track motion and posture changes, thereby aiding in movement artifact identification and reduction.

#### EEG–fNIRS survey results

We investigated the use of auxiliary signals and artifact removal techniques on EEG and fNIRS data, beyond channel pruning, filtering, and normalization, arriving at an evident asymmetrical treatment between modalities (Fig. 4). Out of the 124 studies, 81 of them incorporated artifact removal algorithms for EEG data, versus only 43 for fNIRS. Among the latter, 21 employed motion-artifact removal, 12 tackled physiological confounders, and just 10 of studies handled both type of artifacts. EEG artifact removal was heavily dominated by ICA and its variants (60 articles), used for reducing the effect of cardiac, ocular, and muscular confounding signals, followed by other EOG-regression algorithms, Laplacian filtering for muscle contamination (Fitzgibbon et al., 2013), and Artifact Subspace Reconstruction (ASR) (Chang et al., 2020). For fNIRS, in contrast, the subset of articles that did apply noise canceling methods follows a more uniform distribution compared with EEG, signaling a lack of consensus on “best practices” for removing artifacts (Yücel et al., 2021, 2024). For motion artifacts, PCA, wavelet filtering, spline interpolation, and Temporal Derivative Distribution Repair (TDDR) (Fishburn et al., 2019) were the preferred approaches, while GLM and SS channel subtraction were typically utilized to remove physiological noise. The category “Others” encompasses methods used only once, such as Chauvenet’s criterion (T. Talukdar et al., 2013), Savitzky-Golay filtering (Jahani et al., 2018), spatial filtering, and Variational Mode Decomposition (VMD) (Hossain et al., 2022).

Regarding auxiliary signals, we found that 81 articles did not incorporate any of them, 36 included EEG-related ones, such as EOG and EMG, and only 12 recorded fNIRS-specific signals, such as SS channels and SpO_2_. In some cases, auxiliary signals were incorporated during single-modality preprocessing, and in others they entered as inputs of decision-level algorithms on the same footing as the neuroimaging recordings.

#### 3.2.2 EEG-DOT survey results

From the five surveyed EEG–DOT fusion articles, two of them used simulated data and one did not eliminate artifacts nor included auxiliary signals. Chalia et al. (2016) included ECG monitoring to remove EEG artifacts manually via a clinical neurophysiologist, and DOT motion artifacts were handled by combining visual inspection and the Homer2 toolbox (T. J. Huppert et al., 2009). Cao et al. (2023) used ICA with PCA for EEG denoising, and TDDR with GLM (incorporating recorded gyroscope and accelerometer signals as regressors) to remove motion-induced and physiological noise from DOT data.

### 3.3 EEG-informed fNIRS analysis

**Fig. 5.**
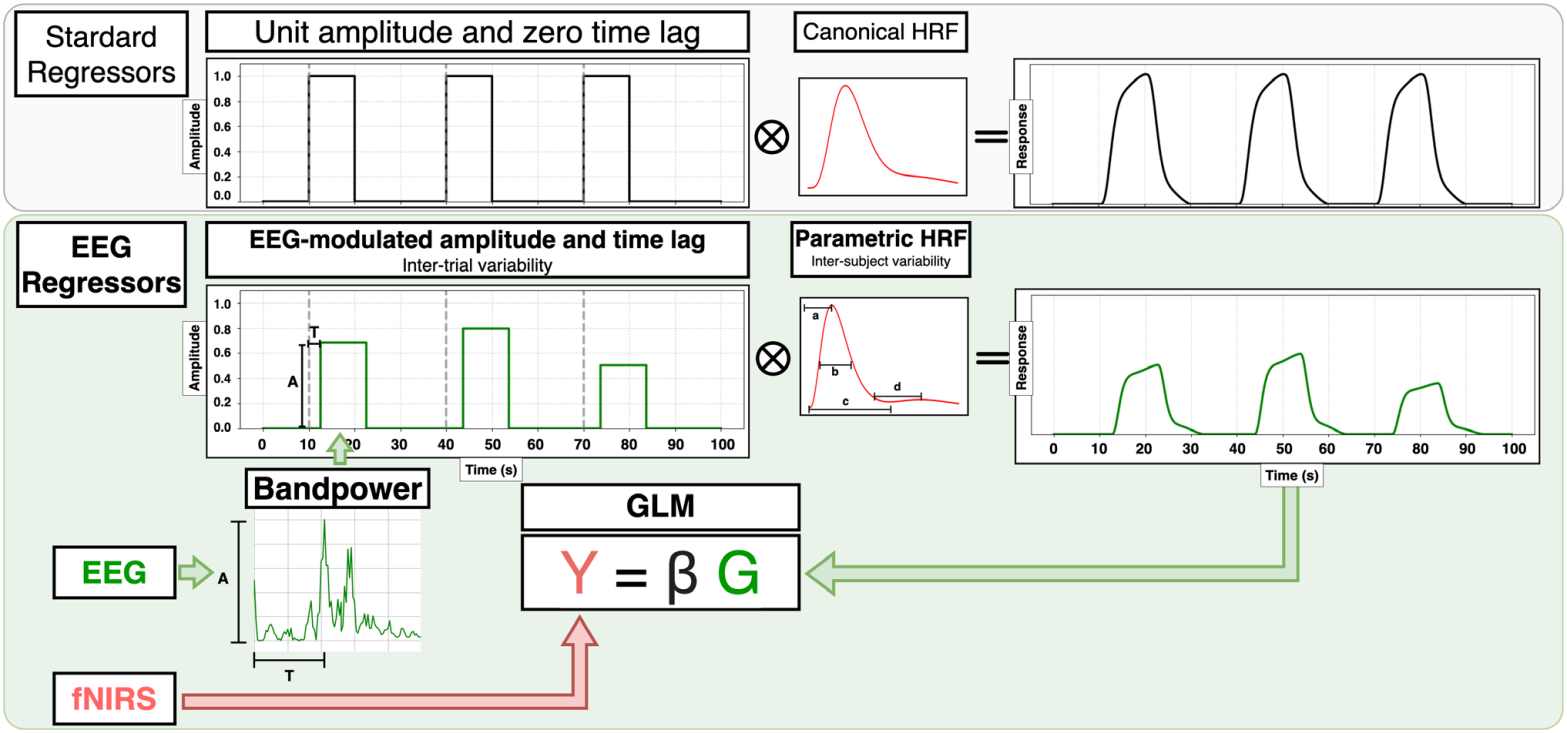
GLM fNIRS analysis with standard regressors (top) and one of its EEG-informed variants (bottom). In the latter, the peak (A) and delay (T) are derived from frequency-specific EEG bandpower signals and used to scale and shift the standard boxcar function of each trial. Additionally, the canonical HRF is replaced by a function with subject-specific parameters, such as time-to-peak (a) or time-to-undershoot (c). The resulting hemodynamic response design matrix (G) captures trial-to-trial and inter-subject variability.

Some EEG–fNIRS fusion techniques treat modalities in asymmetrical ways, leveraging one modality to inform the other, therefore, significantly enhancing signal quality and interpretability compared with non-directional fusion or single-modality approaches. A drawback of these unilateral approaches is that the former modality does not enjoy the benefits of multimodal fusion. In EEG-informed fNIRS analysis, EEG features are utilized to guide or restrict fNIRS processing in order to improve the quality of the reconstructed hemodynamic response. One of the most successful implementations of the EEG-informed methods consists of using EEG features to build regressors for an fNIRS GLM (Fig. 5), where fNIRS hemodynamic signals (HbO and HbR) are treated as the dependent variable (Peng et al., 2014; Pouliot et al., 2014).

#### 3.3.1 fNIRS GLM analysis with EEG-derived regressors

##### fNIRS GLM analysis

The GLM is a widely used technique for fNIRS analysis. Denoting number of fNIRS channels and sample points by $N_{y}$ and T, respectively, GLM expresses the measured oxygenated or deoxygenated hemodynamic signal, $Y\in {\mathbb{R}}^{N_{y}\times T}$ , as a linear combination of the predicted hemodynamic responses, additional confounding regressors, and a residual

$$\text{Y}=\beta \text{G}+\text{E},\underset{\text{OLS estimate}}{\to }\hat{\beta }=\text{Y}{\text{G}}^{⊤}{\left(\text{G}{\text{G}}^{⊤}\right)}^{-1}.$$ (1)

Here, $\text{E}\in {\mathbb{R}}^{N_{y}\times \;T}$ is the residual error matrix, and $\beta \in {\mathbb{R}}^{N_{y}\times \;K}$ are the to-be-determined coefficients of the K regressors encoded in the rows of the design matrix $\text{G}\in {\mathbb{R}}^{K\times \;T}$ . The latter can be divided into task/condition-specific evoked hemodynamic responses, and nuisance regressors utilized for removing physiological and motion artifacts, such as polynomial drifts and features derived from auxiliary signals. In the GLM standard formulation (Diamond et al., 2006; Kirilina et al., 2013), the hemodynamic response is modeled as the convolution between a canonical hemodynamic response function (HRF) and a boxcar function. The HRF parameterizes the hemodynamic shape in response to a short stimulation and is typically modeled as combination of gamma-variant (A. F. Abdelnour & Huppert, 2009; Glover, 1999) or Gaussian functions. The boxcar function encodes the temporal structure of the corresponding task-evoked stimuli.

The optimal coefficients $\hat{\beta }$ can be estimated in various forms. Assuming uncorrelated residual, and a non-singular $\text{G}{\text{G}}^{T}$ matrix (implying, in particular, T≥K ), the Ordinary Least Squares (OLS) estimate (1) exists and is unique. Other alternative estimates of $\hat{\beta }$ include regularization terms, such as Ridge regression to handle collinearity, or iterative re-weighting techniques to deal with outliers and correlated noise, such as Iteratively Reweighted Least Squares (IRLS) (Holland & Welsch, 1977) and Autoregressive IRLS (AR-IRLS) (Barker et al., 2013), which extends IRLS by prewhitening with an AR noise model. Ultimately, statistical tests, such as t-test, can be performed on $\hat{\beta }$ to identify channels encoding statistically significant differences among conditions.

##### GLM with EEG-derived regressors

In the simplest GLM formulation, the same HRF shape and boxcar function are assumed across brain regions, subjects, and trials (Fig. 5 top). While this approximation to a stationary brain response makes the algorithm simpler, in reality these variabilities are present (Handwerker et al., 2004; T. Huppert et al., 2006), and so the method may not accurately describe the underlying cerebrovascular activity. Parametric modeling of HRFs and the introduction of EEG-derived boxcar functions have proven to be promising modifications to achieve more favorable performance (Fig. 5 bottom). Features from frequency-specific time-varying EEG bandpower, such as peak and latency with respect to stimulus onset, can be used for the latter purpose (Y. Gao, Jia, et al., 2023). Specifically, the peak delay shifts the start time of the boxcar, and the peak value scales its amplitude. This approach is motivated by the well-established fact that EEG activity systematically changes with specific stimuli, observed either as phase-locked responses (event-related potentials, ERPs) or as induced modulations of oscillatory power (event-related desynchronization/synchronization, ERD/ERS) (Pfurtscheller & Lopes Da Silva, 1999). On top of that, the canonical HRF can be replaced by a parametric double gamma function, optimized with subject-specific parameters, such as time-to-peak or time-to-overshoot (J. Lin et al., 2024).

#### 3.3.2 Survey results

##### GLM-based

From the DOT-EEG survey, we found no articles following the EEG-informed strategy, while the fNIRS–EEG survey returned 12 works (9.6%) (Fig. 2), in which 9 of them implemented similar GLM-based frameworks to the one presented above (Table 1). The first studies to introduce the EEG-informed idea for fNIRS analysis were Peng et al. (2014, 2016) and Pouliot et al. (2014), with the purpose of studying brain activity during epileptic events. There, onset of seizures or interictal epileptiform discharges (IEDs or spikes) were marked manually from EEG time traces by professionals and then convolved with canonical HRFs to generate the GLM regressors. In Y. Gao, Jia, et al. (2023), the same EEG bandpower peak-and-latency strategy mentioned above was used. After regression, the fitted HbO and HbR signals were passed through a Common Spatial Pattern (CSP) algorithm (Y. Wang et al., 2005) and concatenated with other EEG-derived CSP features to test the performance of a hybrid BCI system on a motor imagery classification task.

**Table 1.** Surveyed studies that implemented GLM-based EEG-informed methods.

Reference	Boxcar stimulus	HRF function	Study objective
Peng et al. (2014)	IEDs (spikes)	Canonical	Epileptic events
Pouliot et al. (2014)			
Peng et al. (2016)			
Y. Gao, Jia, et al. (2023)	Bandpower		Motor Imagery
R. Li, Zhao, et al. (2020)			NVC investigation
Chiarelli et al. (2021)	Bandpower + PCA		FCA in AD
Zama et al. (2019)	ERS/ERD		Response during movement
M. T. Talukdar et al. (2015)	Envelope	Parametric	Median nerve stimulation
J. Lin et al. (2024)	Tensor decomposition		NVC investigation

##### Another EEG-informed variant

On top of these GLM-based methods, M. J. Khan et al. (2018) and Arif et al. (2022) implemented a novel EEG-informed fNIRS analysis approach for the early detection of hemodynamics responses in hybrid BCI systems by analyzing the trajectory of the v= (HbO, HbR) vector in a phase diagram. There, two circles are drawn in the phase diagram: a bigger one corresponding to the maximum magnitude of v during previous resting-state time and a smaller one corresponding to its maximum magnitude during a 1-second window in which the EEG signal was active. Positive activity is inferred by identifying certain patterns of the vector’s trajectory with respect to the inner and outer circles. With this vector-phase analysis, M. J. Khan et al. (2018) report not only an improved classification accuracy over their single-modality counterparts, but also an impressive reduction of command-generation time to 1.5 seconds on average. Another EEG-informed fusion method based on regularized CSP theory, called R-CSP-E, was proposed in Y. Wang et al. (2022), in which the weighted covariance matrix was transferred from EEG to fNIRS. Fusion occurs when computing the spatial filter for fNIRS in the CSP algorithm, in which the standard fNIRS covariance matrix is replaced by a regularized weighted average between the covariance matrices of each modality. The filter features were then used in a K-nearest neighbors (KNN) classifier.

### 3.4 fNIRS-informed EEG analysis

**Fig. 6.**
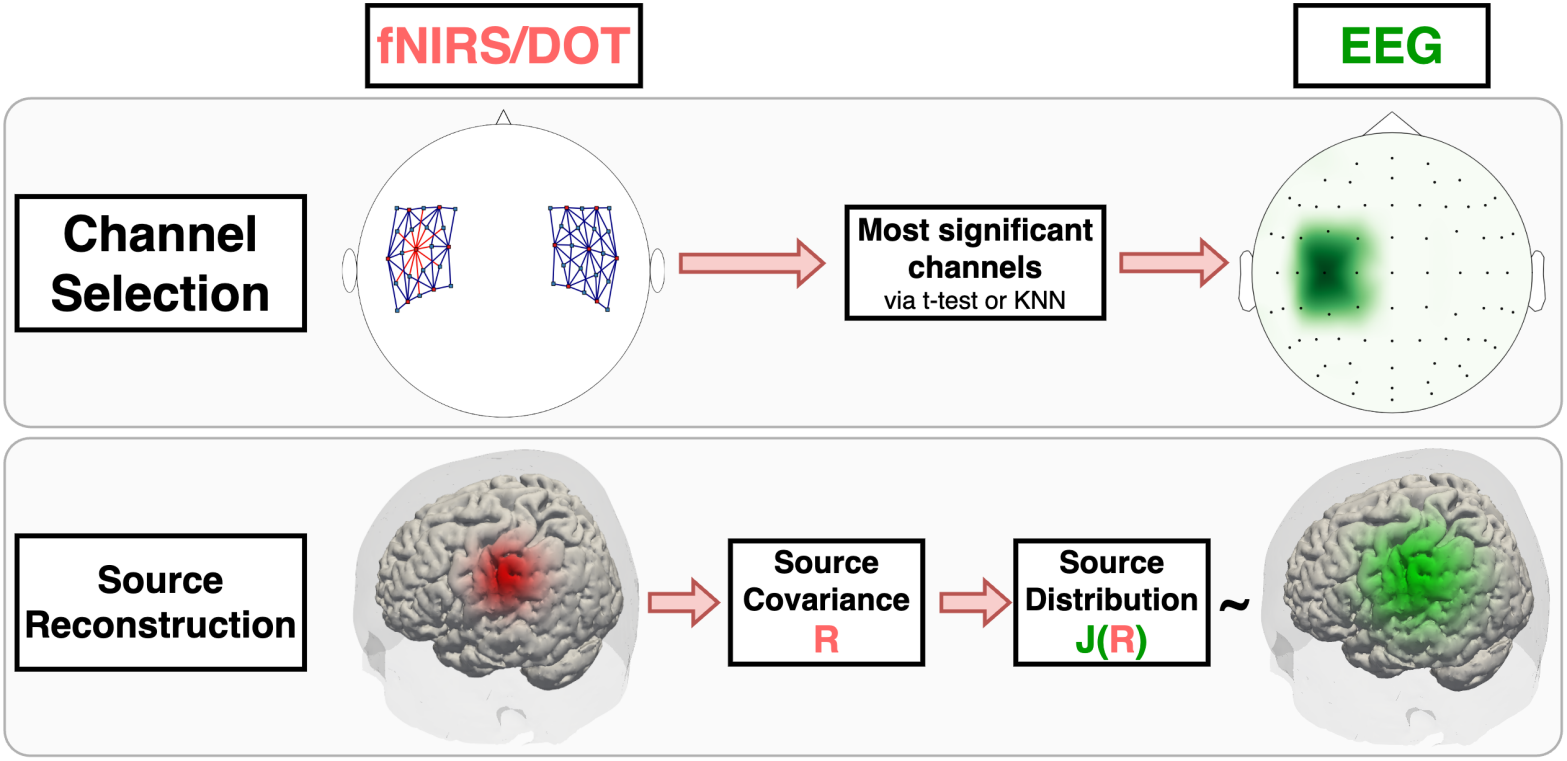
fNIRS/DOT-informed fusion approaches: channel selection and source reconstruction.

Using fNIRS features during EEG data analysis is another asymmetrical fusion technique. These methods leverage fNIRS spatial resolution in order to guide EEG processing, and are typically applied in two contexts: channel selection and source imaging analysis (Fig. 6). The former is commonly found in single-trial analysis, where the experimental paradigm involves different conditions or tasks, and its main goal is to reduce the dimensionality of the problem without compromising performance. This algorithm selects the subset of fNIRS channels that yields the highest contrast between conditions, according to some statistical analysis, such as t tests (R. Li et al., 2017), and then, the closest EEG channels to these fNIRS channels are selected.

#### 3.4.1 fNIRS-informed EEG source imaging analysis

##### EEG image reconstruction

EEG source imaging corresponds to the problem of, given some recorded electrical activity on the scalp, reconstructing the cortical sources that generated that signal. This inverse problem is inherently ill-posed due to the fact that several source configurations can lead to the same scalp data. Nevertheless, source reconstruction algorithms can make certain assumptions on the underlying sources in order to mitigate this problem and hence improve spatial resolution. The linear forward model is one of the most popular proposals (Haufe et al., 2014; Parra et al., 2005):

$$\text{x}(t)={\text{G}}_{\text{e}}\text{j}\left(t\right)+\epsilon (t).$$ (2)

Here, $x(t)\in {\mathbb{R}}^{N_{x}\times \;1}$ encodes the EEG recordings for the $N_{x}$ channels, and $\epsilon (t)\in {\mathbb{R}}^{N_{x}\times \;1}$ is a noise term. The underlying (unknown) sources are encoded in $\text{j}(t)\in {\mathbb{R}}^{K\times \;1}$ , corresponding to K dipole cortical currents. ${\text{G}}_{\text{e}}\in {\mathbb{R}}^{N_{x}\times \;K}$ is the so-called lead field matrix, which is known, or estimated by a given head model, and parameterizes how strong each dipole signal contributes to each channel. One way of inverting this model (see also Mäkelä et al., 2018; Mosher and Leahy, 1999, for approaches based on the MUSIC algorithms family) is by assuming specific distributions for the noise and currents, such as ϵ∼N(0,C) and j∼N(0,R), where $\text{C}\in {\mathbb{R}}^{N_{x}\times \;N_{x}}$ and $\text{R}\in {\mathbb{R}}^{K\times \;K}$ are the corresponding covariance matrices and N denotes the Gaussian distribution. Under these assumptions, one can obtain the following estimate for the currents:

$$\hat{j}=RG_{e}^{\;\;\;T}{\left(G_{e}RG_{e}^{\;\;\;\;T}+\lambda C\right)}^{-1}x,$$ (3)

where λ is a regularization parameter, representing the trade-off between model accuracy and complexity. The solution (3) can be derived from a Bayesian perspective, where $\hat{j}$ is the maximum a posteriori (MAP) estimate (Sato et al., 2004), corresponding to the current that maximizes the posterior distribution $P(\text{j}\;|\;\text{x})=\frac{P(\text{x}\;|\;\text{j})P\left(\text{j}\right)}{P\left(\text{x}\right)}$, with the likelihood $P(\text{x}\;|\;\text{j})=N({\text{G}}_{\text{e}}\text{j},\text{C})$ , the prior $P\left(\text{j}\right)=N\left(0,\frac{\text{R}}{\lambda }\right),$ and P(x) independent of j. Prior knowledge of the sources is encoded in R, which coincides with the identity matrix when no information is provided. It is worth emphasizing that the same solution (3) can also be reached from a minimum norm approach (Hauk, 2004), where C and R are not associated with specific distributions. Regardless of the exact approach, such a “blind” model is insufficient to resolve the ill-posed inverse problem fully.

##### Using fNIRS data as prior

A promising direction to overcome these limitations is to use data from other modalities with better spatial resolution as priors, such as fMRI or fNIRS (Fig. 6). In particular, two proposals created in the context of MRI–MEG/EEG fusion have been adapted for fNIRS–EEG fusion: Variational Bayesian Multimodal EncephaloGraphy (VBMEG) (Sato et al., 2004) and Dynamic Brain Transition Network (DBTN) (Nguyen et al., 2016). In VBMEG, anatomical information is used to determine the locations and orientations of the dipole currents, while functional information, from fMRI or fNIRS, is used to estimate the current amplitudes. More precisely, VBMEG assumes a diagonal covariance matrix for the currents $\text{R}=\text{diag}(r_{1},r_{2},\ldots ,r_{K})$ , with the current variances $r_{2},\ldots ,r_{K}$ following parametric Gamma distributions. Among the distributions’ free parameters, the mean variance values, which control the intensity of the corresponding currents, are estimated from fMRI or fNIRS features. In contrast, DBTN models R as the sum of P covariance components ${\text{Q}}_{\text{i}}={\text{q}}_{\text{i}}{\text{q}}_{\text{i}}^{⊤}\text{i}=1,\ldots ,P$ , where $q_{i}$ are K-dimensional vector maps of the voxels that show statistically significant activity between experimental conditions (e.g., task vs. baseline). These maps are calculated via a GLM analysis on fMRI or fNIRS data.

#### 3.4.2 Survey results

##### Channel selection

We encountered 8 articles (6.4%) implementing fNIRS-informed EEG analysis methods, 3 (2.4%) for channel selection, and 5 (4%) for source imaging analysis (Table 2). With the purpose of increasing motor task classification accuracy of hybrid BCI systems with as few channels as possible, R. Li et al. (2017) selected one fNIRS channel per hemisphere based on GLM analysis and t-tests, and Moslehi and Davies (2021) applied the ReliefF algorithm (Urbanowicz et al., 2018) (a filter-based method using KNN to weight channel relevance) to pick either the top five or three fNIRS channels from each frontal and motor regions. In both studies, the adjacent EEG channels to the selected fNIRS ones were chosen.

**Table 2.** Surveyed studies that implemented fNIRS/DOT-informed methods.

Approach	Reference	METHOD	Study objective
Channel Selection (fNIRS)	Al-Shargie et al. (2016)	t-test	Mental stress assessment
	R. Li et al. (2017)		BCI classification
	Moslehi and Davies (2021)	ReliefF algorithm	
Source Reconstruction (fNIRS)	Aihara et al. (2012)	VBMEG	Reconstruction methods comparison
	Morioka et al. (2014)		BCI classification
	R. Li et al. (2019)	DBTN	FCA in AD
	R. Li, Li, et al. (2020)		FCA in poststroke patients
	X. Li et al. (2022)		
Source Reconstruction (DOT)	Cao et al. (2021)	ReML	Method presentation
	Cao et al. (2023)		Method validation (visual task)
	Qin et al. (2024)		Method validation (auditory task)

##### fNIRS-informed source imaging

For source imaging analysis, Aihara et al. (2012) were the first to study on incorporating the idea of fNIRS-informed EEG source reconstruction, by using the VBMEG algorithm introduced above. There, the authors compared the performance of VBMEG for estimating cortical currents with fMRI prior, fNIRS prior, and no prior, on subjects performing a finger-tapping task. Using HbO peak values for modeling current mean variances, they concluded that the novel Bayesian technique allows to obtain relatively high spatiotemporal, physiologically plausible, brain activity, which cannot be obtained using EEG data alone. The same VBMEG method was applied later in Morioka et al. (2014) for BCI applications, where subjects’ left or right spatially attended direction was classified. This time, the current mean variances were estimated from HbO t-statistics between attention and control conditions. The DBTN method was utilized in a series of articles by R. Li et al. (2019), all of them following a similar approach in which the reconstructed cortical currents were first grouped into Regions of Interest (ROI) and then fed into a weighted Phase Lag Index (wPLI) algorithm (Vinck et al., 2011) to characterize brain connectivity networks in AD (R. Li et al., 2019), and poststroke patients (R. Li, Li, et al., 2020; X. Li et al., 2022).

##### DOT-informed source imaging

In the context of DOT–EEG fusion, Cao and collaborators proposed an algorithm that utilizes DOT reconstruction as the spatial prior of EEG reconstruction using the restricted maximum likelihood (ReML) framework (F. Abdelnour et al., 2010). There, the same EEG linear model (2) is used, together with a small extension of the current estimates (3), in which additional covariance-dependent terms are added. As for fNIRS-informed EEG source imaging, DOT priors enter through R, which in this case is assumed to be diagonal with the non-zero entries related to the reconstructed HbO activities from DOT data. This relationship guarantees that a brain voxel should only have high electrical activity (reconstructed from EEG) when the hemodynamic activity (reconstructed from DOT) is high. Cao et al. (2021) introduced the framework and demonstrated its high spatiotemporal resolution for source recovery on simulated data. More precisely, they showed that neuronal sources that are spatially (2.3–3.3 cm) and temporally (50 ms) close can be clearly distinguished by the fusion method, but not by either modality in isolation. Moreover, they showed that such spatial resolution can be achieved even in DOT systems with a sparser, non-high-density optode configuration. The authors also compared the performance with the fNIRS-informed method of Aihara et al. (2012), concluding that when fNIRS projection (instead of DOT reconstruction) is used as the spatial prior, the algorithm performance drops. Later, Cao et al. (2023) validated the high spatiotemporal resolution of the ReML-based algorithm on a real-world dataset with subjects undergoing a visual stimulus task, in which the stimulus speed was faster than the temporal resolution of DOT. Finally, Qin et al. (2024) utilized the same framework for image reconstruction on simulated auditory cortex data, but this time including a novel data-informed method to select optimal DOT channel lengths for a more accurate source depth reconstruction.

### 3.5 Model-based EEG–fNIRS symmetric methods

**Fig. 7.**
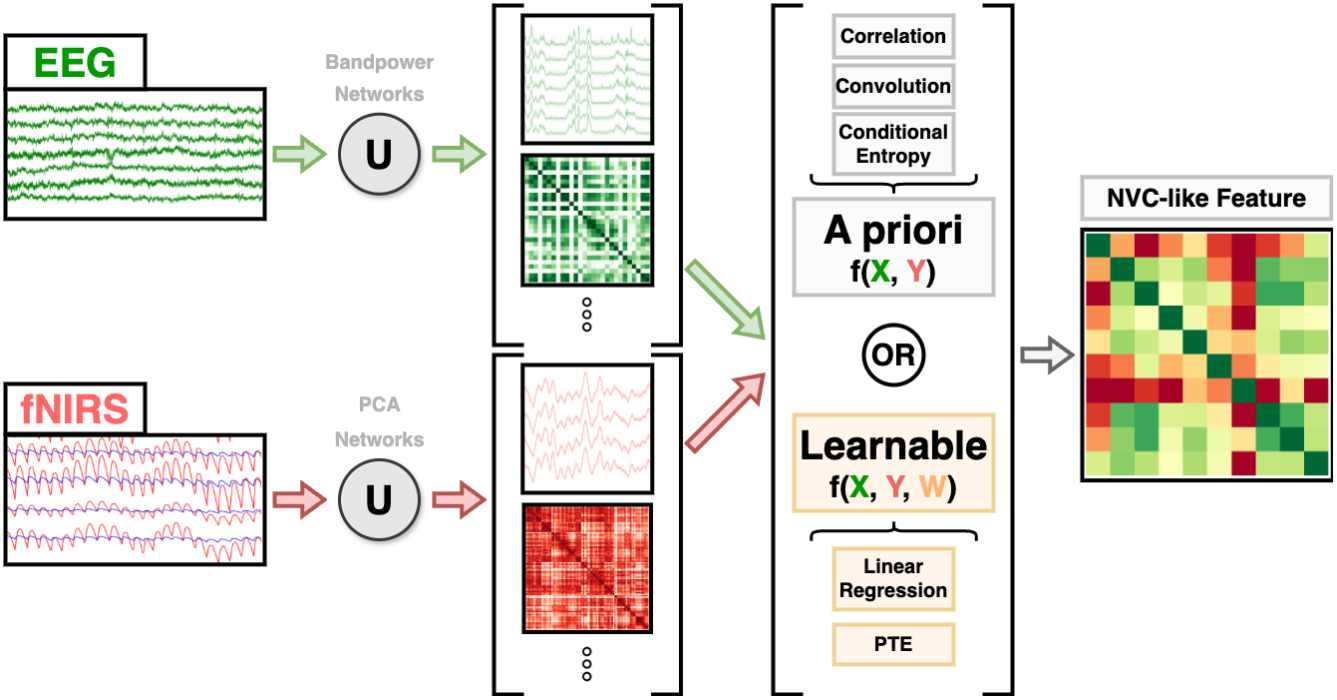
A priori and learnable model-based symmetric fusion strategies.

The methods reviewed so far leverage the strengths of one modality to inform the other. While these methods have been successfully applied to capture inter-trial variability (Section 3.3.1) or achieve higher spatiotemporal resolution than each individual modality (Section 3.4.1), the asymmetrical treatment makes one of the modalities lack the benefits of the fusion. Symmetric fNIRS–EEG analysis methods represent an integrative approach where both modalities contribute equally to the fusion process. In particular, model-based symmetric fusion (Fig. 7) relies on knowledge to explicitly model relationships between neural activity and each of the recorded signals, leveraging physiological principles to guide the fusion process. These methods typically include a single-modality preprocessing step before fusion to bring the datasets to a common feature space, such as bandpower time courses, or network connectivity matrices.

Some of the less sophisticated approaches consist of NVC-like feature generation employing handcrafted functions to merge single-modality data, such as correlation, wavelet coherence, or convolution. While these methods utilize knowledge from the NVC theory to build non-linear combinations of modalities, they lack tunable parameters that could improve performance upon training.

#### 3.5.1 Survey results

##### fNIRS

From the fNIRS–EEG survey, we found 29 articles (23.2%) adopting a model-based symmetric framework (Table 3). Among them, only 4 articles (3.2%) used model-based symmetric methods with learnable parameters. In Sood et al. (2016), the authors developed and tested a Kalman-Filter-based online parameter estimation of an autoregressive (ARX) model to track the transient coupling relation between EEG bandpower changes and fNIRS HbO changes signals during transcranial Direct Current Stimulation (tDCS). There, fusion takes place upon using the EEG and fNIRS features as inputs and outputs of the ARX model, respectively. A novel permutation transfer entropy (PTE) model was proposed in Y. Gao, Liu, et al. (2023) borrowing some ideas from the GLM-based EEG-informed algorithm described in Section 3.3. There, EEG features, corresponding to the peak and latency of the first principal components of EEG frequency bands, are used to modify a typical boxcar function. The latter is then convolved with a canonical HRF to build a design matrix. As reviewed in Section 3.3.1, this matrix is typically used for modeling the hemodynamic response in an fNIRS GLM. However, here PTE is used instead, to consider the directed coupling between the neuronal activities and hemodynamic response. Finally, the generalized relative entropy of PTE time points is computed and used as input of a SVM classifier to quantify mental workload. As opposed to the asymmetrical GLM-based EEG-informed approach, here modalities are treated in a symmetrical fashion.

**Table 3.** Surveyed studies that utilized symmetric model-based fusion approaches.

Approach	Reference	METHOD	Study objective
A priori (fNIRS)	L.-C. Chen et al. (2015)	Correlation	NVC investigation
	Keles et al. (2016)		
	Balconi and Vanutelli (2016)		
	Zich et al. (2017)		
	Suzuki et al. (2018)		
	Y. Chen et al. (2020)		
	Saricaoglu et al. (2025)		
	J. Lin et al. (2023)		
	Aghajani et al. (2017)		BCI classification
	Omurtag et al. (2017)		
	C.-T. Lin et al. (2020)		
	Chu et al. (2022)		
	Borgheai et al. (2019)		NVC in ALS patients
	Kaga et al. (2020)		NVC in ADHD patients
	C. Gao et al. (2024)		NVC of myopic subjects
	Y. Zhang and Zhu (2019)		Resting-state FCA
	Nair et al. (2021)		Anesthesia monitoring
	Si et al. (2024)		Emotion assessment
	Qu et al. (2025)		FCA in stroke patients
	Govindan et al. (2016)	Spectral coherence	NVC investigation
	Perpetuini et al. (2020)	Conditional entropy	Working memory in AD
	Nourhashemi et al. (2020)	Transfer entropy	NVC in neonates
	Othman et al. (2021)	Wavelet coherence	Brain injury
	Charly and Onabid (2024)	Convolution	Speech quality
	Wan et al. (2024)	Multimodal covariance network	FCA in autistic patients
A priori (DOT)	Giacometti and Diamond (2014)	Correlation	NVC investigation
	Chalia et al. (2016)		Burst suppression
Learnable (fNIRS)	Sood et al. (2016)	Kalman filter + ARX model	tDCS
	Gentile et al. (2020)	Linear regression	Laser-evoked potential
	Y. Gao, Liu, et al. (2023)	PTE	Mental workload
	T. Gao et al. (2025)	Linear regression	Etomidate use disorder

##### DOT

From the DOT-EEG survey, two works used plain correlation as the fusion strategy. In Giacometti and Diamond (2014), the authors studied the relationship between colocalized HD EEG and fNIRS recordings on a single subject by calculating correlation between EEG and fNIRS cortical sensitivity matrices, and tabulated the maximum correlated pairs to be used for future studies on NVC. Correlations between the inverse models were also computed for the whole cortical surface and for ROI. A qualitative analysis between EEG and DOT data was carried out in Chalia et al. (2016) to study the hemodynamic response to burst suppression on infants with hypoxic ischemic encephalopathy. There, the authors managed to identify a temporal correlation between EEG bursts and HbO and HbR changes in channel and image space.

### 3.6 Data-driven EEG–fNIRS symmetric methods

Fusion methods in this section combine the strengths of symmetric and data-driven approaches to create a flexible framework in which relationships between fNIRS and EEG signals are inferred directly from the data, and used in a bidirectional fashion so each modality receives and provides guidance during processing. We consider these methods to be particularly promising for continuous, naturalistic brain imaging, as their inherent flexibility enables adaptive handling of multimodal data in dynamic and unconstrained settings. Despite these advantages, previous reviews have overlooked these methods. Therefore, this section represents the core contribution of our manuscript, aiming to address this gap and thoroughly explore the potential and characteristics of these underrepresented, yet highly promising, fusion techniques.

#### 3.6.1 Data selection

These algorithms can be further divided into subcategories such as feature/data-selection and feature-extraction. The former category encompasses methods in which a subset of the most relevant EEG and fNIRS features (or raw channel signals) are selected according to some criterion, such as correlation, statistical tests, or mutual information (Table 4). These methods tend to be computationally cheap, and can be used at data/sensor-level (channel selection) or feature-level (feature selection), typically in combination with other feature-extraction algorithms. However, as individual fusion methods, they may fail to uncover complex structures on the data since they do not modify the original signals.

**Table 4.** Surveyed studies that implemented data-driven symmetric methods.

Approach	Reference	METHOD	Study objective
Data Selection	Hasan et al. (2020)	Correlation (channel selection)	BCI classification
	M. U. Khan and Hasan (2020)		
	Ali et al. (2024)		
	Zennifa et al. (2018)	Correlation (feature selection)	Engagement recognition
	Borgheai et al. (2024)		BCI performance prediction
	Yin et al. (2015)	Mutual information	BCI classification
	Deligani et al. (2021)		
	Hosni et al. (2022)	LASSO*	
	Yi et al. (2023)		Depression assessment
	M. Meng et al. (2021)	Mutual information + LASSO	BCI classification
	Ergün and Aydemir (2023)	Cross validation	
	Ge et al. (2019)	ReliefF algorithm	NVC in action observation
	Cicalese et al. (2020)	Correlation (wrapper-based)	AD stages classification
	Ali et al. (2023)	E-WOA	Method presentation
Source Decomposition	Al-Shargie et al. (2017b)	CCA	Mental stress assessment
	Ghasimi and Shamekhi (2024)		Cognitive workload assessment
	Rezaee et al. (2021)	tCCA	tDCS response in stroke patients
	Walia et al. (2022)		Error-related brain states
	Dashtestani et al. (2022)	ssmCCA	Action–observation network
	Su et al. (2023)		NVC during motion
	Dähne et al. (2013)	mSPoC	Method presentation
	Ieong et al. (2019)		NVC in opiate addiction
	Al-Shargie et al. (2016)	jICA	Mental stress assessment
	Hossain et al. (2022)	WPD-CCA	Motion artifact correction
	Xu et al. (2023)	PCA	BCI classification
	Ran et al. (2025)	TdCCA	FCA in Moyamoya disease
	Nia et al. (2025)	CCA + kCCA	Emotion recognition
	McLinden et al. (2024)	PAC	NVC investigation
	Zhu et al. (2024)	CMVGP-CCM	
	Hou et al. (2024)	Hybrid brain network	Emotion recognition
	Blanco et al. (2024)	Multiplex network	FCA

* Least Absolute Shrinkage and Selection Operator algorithm (Hastie et al., 2015).

#### 3.6.2 Source-decomposition methods

**Fig. 8.**
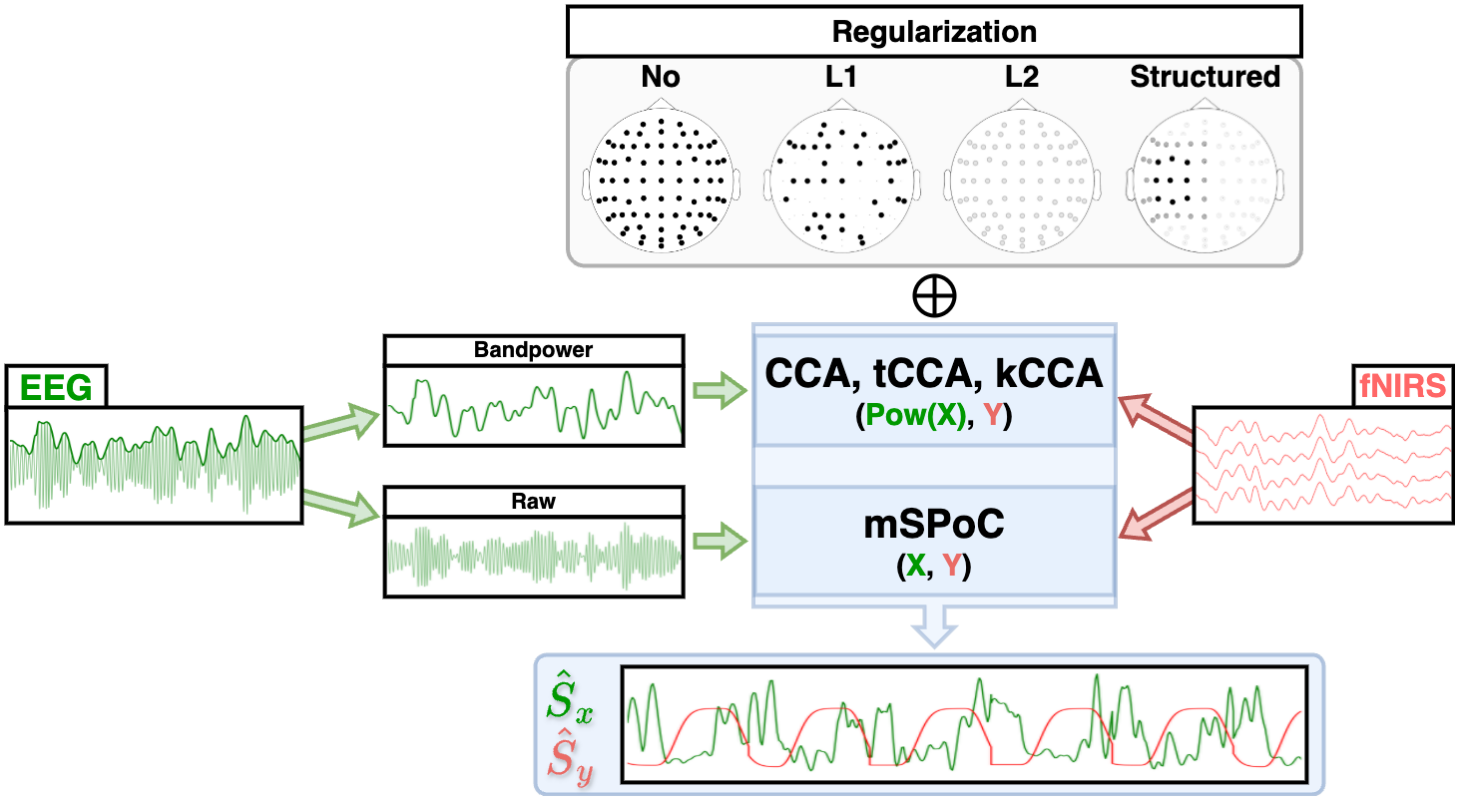
Multimodal source-decomposition methods.

Data-driven feature extraction combines EEG and fNIRS data at sensor level, and are promising alternatives to their model-based counterparts when theoretical models are insufficient to capture the complex dynamics of the underlying physiological process. Multimodal source decomposition (Zhuang et al., 2020) (Fig. 8) is a popular approach in which the data are assumed to follow a linear forward model:

$$x\left(t\right)={\text{A}}_{x}{\text{s}}_{x}\left(t\right)+\epsilon_{x}\left(t\right),\;\;\text{y}\left(t\right)={\text{A}}_{y}{\text{s}}_{y}\left(t\right)+\epsilon_{y}\left(t\right).$$ (4)

Here, datasets are encoded in $\text{X}\in {\mathbb{R}}^{N_{x}\times \;T_{x}}$ and $\text{Y}\in {\mathbb{R}}^{N_{y}\times \;T_{y}}$, and their columns $\text{x}\left(t\right)\in {\mathbb{R}}^{N_{x}\times \;1}$ and $\text{y}\left(t\right)\in {\mathbb{R}}^{N_{y}\times \;1}$ denote multivariate EEG and fNIRS observations. $N_{x},N_{y}$ and $T_{x},T_{y}$ are the number features and samples/observations, respectively.^6^ Measurements are decomposed into components or sources $s_{x}\in {\mathbb{R}}^{K_{x}\times \;1},s_{y}\in {\mathbb{R}}^{K_{y}\times \;1}$ , whose strengths are modulated by the corresponding spatial activation patterns, encoded in the columns of ${\text{A}}_{x}\in {\mathbb{R}}^{N_{x}\times \;K_{x}},{\text{A}}_{y}\in {\mathbb{R}}^{N_{y}\times \;K_{y}}$. Despite a similar linear form between (4) and (2), it is worth noting the blind nature of the current source separation model, in which not only the sources but also the mixing matrices ${\text{A}}_{x}$ and ${\text{A}}_{y}$ are unknown and must be estimated from the data. The noise terms $\epsilon_{x},\epsilon_{y}$ are supposed to capture the activity which is not explained by the sources. We assume both signals contain a subset $K\leq \text{min}(K_{x},K_{y})$ of components which are related by some unknown relationship. The main objective of source-decomposition methods is to uncover such a relation and find the reconstructed sources via a linear backward model

$${\hat{\text{s}}}_{x}\left(t\right)={\text{W}}_{x}^{⊤}\text{x}\left(t\right),\;\;{\hat{\text{s}}}_{y}\left(t\right)={\text{W}}_{y}^{⊤}\text{y}\left(t\right).$$ (5)

The filters ${\text{W}}_{x}\in {\mathbb{R}}^{K_{x}\times \;N_{x}},{\text{W}}_{y}\in {\mathbb{R}}^{K_{y}\times \;N_{y}}$ can then be used to estimate the spatial patterns of the corresponding forward model via (Haufe et al., 2014)

$${\hat{\text{A}}}_{x}={\text{C}}_{x}{\text{W}}_{x}{\left({\text{W}}_{x}^{⊤}{\text{C}}_{x}{\text{W}}_{x}\right)}^{-1},\;\;{\hat{A}}_{y}={\text{C}}_{y}{\text{W}}_{y}{\left({\text{W}}_{y}^{⊤}{\text{C}}_{y}{\text{W}}_{y}\right)}^{-1},$$ (6)

where ${\text{C}}_{x}$, ${\text{C}}_{y}$ are the covariance matrices for each modality. The filters are obtained as the solution of a given inverse optimization problem, which ultimately defines the specific source-decomposition method. In what follows, we describe the most relevant of these methods extracted from the survey. When required, we assume $T_{x}=T_{y}=T$ , which can be achieved upon resampling.

#### 3.6.3 CCA and its variants

##### Standard CCA

Canonical Correlation Analysis (CCA) (Hardoon et al., 2004; Hotelling, 1936) is a data-driven, unsupervised method that seeks to solve the inverse problem (5) by finding linear combinations of the data that are maximally correlated with each other. In its simplest formulation, CCA finds one-dimensional filters ${\text{w}}_{x}\in {\mathbb{R}}^{N_{x}\times \;1}$ and ${\text{w}}_{y}\in {\mathbb{R}}^{N_{y}\times \;1}$ such that the reconstructed sources (a.k.a canonical variates) ${\hat{s}}_{x}\left(t\right)={\text{w}}_{x}^{⊤}\text{x}\left(t\right),{\hat{s}}_{y}\left(t\right)=$ ${\text{w}}_{y}^{⊤}\text{y}\left(t\right)$ maximize the correlation coefficient $\text{Corr}({\hat{s}}_{x},{\hat{s}}_{y})$ . The optimization problem can be brought to a constrained quadratic form by imposing unit variance on the reconstructed sources

$$\underset{{\text{w}}_{x},{\text{w}}_{y}}{\text{max}}{\text{w}}_{x}^{⊤}{\text{C}}_{xy}{\text{w}}_{y}\;\;\text{s.t.}\;\;{\text{w}}_{x}^{⊤}{\text{C}}_{x}{\text{w}}_{x}={\text{w}}_{y}^{⊤}{\text{C}}_{y}{\text{w}}_{y}=1,$$ (7)

where we denoted the cross-modality covariance matrix ${\text{C}}_{xy}$ . By using the Lagrange-multiplier method (Hardoon et al., 2004), one can reformulate (7) into a generalized eigenvalue problem where eigenvalues coincide with estimated source covariances, $\lambda =\text{Cov}({\hat{s}}_{x},{\hat{s}}_{y})$ , and so the optimal solution corresponds to the one with the largest eigenvalue. It can be shown (L. Sun et al., 2009) that if X,Y are whitened, that is, ${\text{C}}_{x}=\text{I}$ and ${\text{C}}_{y}=\text{I}$ , then CCA reduces to Partial Least Squares (PLS) (Wold et al., 1984; Worsley et al., 1997). The one-unit algorithm can be extended to find $K\leq \text{min}(N_{x},N_{y})$ filters (Kettenring, 1971), ${\text{W}}_{x},{\text{W}}_{y}$, by solving the same generalized eigenvalue problem but for the first K eigenvectors with the highest eigenvalues. Picking an orthonormal basis, which is always possible via a deflation algorithm (Mackey, 2008), for instance, the resulting filters maximizes $\text{Trace}\left\{{\text{W}}_{x}^{⊤}{\text{C}}_{xy}{\text{W}}_{y}\right\}$ such that ${\text{W}}_{x}^{⊤}{\text{C}}_{x}{\text{W}}_{x}={\text{W}}_{y}^{⊤}{\text{C}}_{y}{\text{W}}_{y}=\text{I}$ .

##### Regularized CCA

In modern neuroimaging datasets, we often encounter situations in which the number of variables is much larger than the number of observations, making CCA to suffer from issues such as high-dimensionality, multicollinearity, and overfitting. This is typically the case in group-level analysis, where variables such as voxels or time points clearly exceed the number of subjects (observations). Regularized CCA adds penalty terms to the objective function (7), controlling model’s complexity, reducing overfitting, and improving generalization. These regularized methods admit a general constrained optimization problem description, in terms of generic convex penalty functions $P_{x}({\text{w}}_{x})$ and $P_{y}({\text{w}}_{y})$ (Witten et al., 2009; Witten & Tibshirani, 2009)

$$\begin{array}{l} \underset{{\text{w}}_{x},{\text{w}}_{y}}{\text{max}}{\text{w}}_{x}^{⊤}{\text{C}}_{xy}{\text{w}}_{y}\;\;\text{s.t. }{\text{w}}_{x}^{⊤}{\text{C}}_{x}{\text{w}}_{x}\;={\text{w}}_{y}^{⊤}{\text{C}}_{y}{\text{w}}_{y}\;=1,\;\;P_{x}({\text{w}}_{x})\leq c_{x},\;\;\\ \;\;\;P_{y}({\text{w}}_{y})\leq c_{y}, \end{array}$$ (8)

where $c_{x}$, $c_{y}$ are positive scalars. Many implementations (J. Chen et al., 2013; Dashtestani et al., 2022; Witten et al., 2009) replace the covariance matrices, ${\text{C}}_{x}$ and ${\text{C}}_{y}$, with identity matrices, claiming that there is no loss in performance.

##### ElasticNet CCA

Two of the most common choices for the penalty terms $P_{x}$ and $P_{y}$ are the L2-norm, $P\left(\text{w}\right)=|\left|\text{w}\right|{|}_{2}^{2}$, which regulates the overall magnitude of the coefficients and leads to Ridge CCA (Bie & De Moor, 2003), and the L1-norm, $P\left(\text{w}\right)=\;||\text{w}|{|}_{1}$, which searches for a sparser solution, setting some coefficients to zero, leading to sparse CCA (sCCA) (Parkhomenko et al., 2009). Both penalty terms can be combined to obtain Elastic Net regularization (Hastie et al., 2015).

##### ssCCA

Elastic Net regularization can be modified to include contextual information about the local structure of the dataset, leading to structured sparse CCA (ssCCA). This is particularly relevant in neuroimaging, where features (e.g., channels or brain regions) follow specific spatial distributions. This can be achieved via Graph-constrained Elastic Net (GraphNet), where the L2 norm penalty function $P\left(\text{w}\right)=\;||\text{w}|{|}_{2}^{2}$ is replaced by $P(\text{w})={\text{D}}^{⊤}\text{Lw}$ with L corresponding to the Laplacian matrix of an undirected weighted graph (Chung, 1997). The latter is used as a representation of the dataset, in which features are the nodes, and (weighted) edges indicate which and how features are connected. These edges are encoded in the adjacency matrix $\text{B}\in {\mathbb{R}}^{N\times N}$ with N corresponding to the number of features/nodes. With B, one can build the degree matrix D, which is diagonal with the non-zero entries ${\text{D}}_{nn}=\sum_{m\;=\;1}^{N}{\text{B}}_{nm}$ , and the Laplacian matrix, L=D−B , which is the canonical matrix representation of a graph. GraphNet encourages filter components $w_{n}$ to be more similar, if their corresponding features are more connected (He & Niyogi, 2003). The latter is regulated by the weights in B, which need to be determined a priori. Popular choices are the binary KNN adjacency matrix, where each feature is connected to its k closest neighbors with a value of 1, or the distance-based weighted approach, where closer features get assigned higher weights using functions such as Gaussian kernels. In the context of wearable neuroimaging, the montage layout/grid can be used to define such connections. The constrained optimization problem defining ssCCA can be also brought into the form of (8) (J. Chen et al., 2013; Witten et al., 2009), with $P_{x}({\text{w}}_{x})={\text{w}}_{x}^{⊤}{\text{L}}_{x}{\text{w}}_{x}$ and $P_{y}({\text{w}}_{y})={\text{w}}_{y}^{⊤}{\text{L}}_{y}{\text{w}}_{y}$, in which ${\text{L}}_{x}$ and ${\text{L}}_{y}$ are the Laplacian matrices of each dataset.

##### kCCA

Linear models (4), such as standard CCA, are reasonable first-order approximations for many neuroimaging applications, but they may fall short in certain scenarios where the modalities’ relationships are non-linear in nature. Kernel CCA (kCCA) (Friston et al., 2000; Fukumizu & Gretton, 2007) addresses this limitation by implicitly mapping the original datasets into a high-dimensional feature space, X→Φ(X),Y→Φ(Y), embedded in kernel functions ${\text{K}}_{x}\equiv \Phi {\left(\text{X}\right)}^{⊤}\Phi (\text{X})\in {\mathbb{R}}^{N_{x}\times \;N_{x}},$ ${\text{K}}_{y}\equiv \Phi {\left(\text{Y}\right)}^{⊤}\Phi (\text{Y})\in {\mathbb{R}}^{N_{y}\times \;N_{y}}$ which are independent of the new number of features. Nonlinearities are encoded in the choice of such kernels, such as polynomials or radial basis (Gaussian) functions, and standard CCA can be recovered by choosing Φ to be the identity function (i.e., Φ(X)=X ). The kCCA algorithm can be obtained as the non-linear extension of (7)

maxwx,wywx⊤KxKywy  s.t.  wx⊤Kx2wx=wy⊤Ky2wy=1. (9)

Interestingly, a similar close-form solution via a generalized eigenvalue problem exists in this case, and the same regularization techniques mentioned above can be applied. By leveraging kernels, kCCA can effectively uncover complex, nonlinear associations between modalities. The need for a pre-defined kernel function to model nonlinearities (model-based) is alleviated in the DL extension of CCA, deep CCA (Andrew et al., 2013), which learns the feature mapping directly from data (data-driven), offering a more flexible and robust approach. However, interpretability in these deep methods is a matter of ongoing research.

##### tCCA

All the methods described so far assume modalities correlate instantaneously, which is certainly not the case in fNIRS–EEG fusion, for instance, where the common underlying sources are time-shifted due to non-instantaneous and non-constant couplings between modalities. Temporally embedded CCA (tCCA) (Bießmann et al., 2010) captures such temporal offsets. While effective, the idea is rather simple: assuming Y is the “delayed” modality, X is time embedded by concatenating time-shifted copies along the feature dimension via $\tilde{\text{X}}\equiv [\text{X}(\tau_{1}),\ldots ,\text{X}(\tau_{P})]\in {\mathbb{R}}^{PN_{x}\times \;T}$ , where $\text{X}(\tau_{i})$ is a copy of the original X but time shifted by $\tau_{i}$. These P time lags must be preselected, and the time dimension size T can be preserved upon padding. Standard CCA (or any of the variants explored above) is then applied to $\tilde{\text{X}}$ and Y, yielding temporal filters that capture delayed correlations between modalities. In the context of fNIRS, this method has been shown to improve performance in HRF recovery in combination with a multimodal extension of the GLM (von Lühmann, Li, Müller, et al., 2020).

##### mCCA

Multiset CCA (mCCA) extends CCA to more than two datasets (Correa et al., 2010; Kettenring, 1971). Given D datasets, ${\text{X}}_{i}\in {\mathbb{R}}^{N_{i}\times \;T},i=1,\ldots ,D$ , mCCA aims at finding D filters ${\text{w}}_{i}\in {\mathbb{R}}^{N_{i}\times \;1}$ such that the overall correlation between datasets is maximized

$$\underset{{\text{w}}_{1},{\text{w}}_{2},\cdots ,{\text{w}}_{D}}{\text{max}}J({\text{w}}_{i}^{⊤}{\text{C}}_{ij}{\text{w}}_{j})\;\;\text{s.t. }\;{\text{w}}_{i}^{⊤}{\text{C}}_{i}{\text{w}}_{i}=1\;\;i,j=1,\ldots ,D\;i\neq j.$$ (10)

Here, ${\text{C}}_{ij}$ is the cross-covariance matrix between datasets ${\text{X}}_{i}$ and ${\text{X}}_{j}$, and ${\text{C}}_{i}$ the covariance of dataset ${\text{X}}_{i}$. J is an appropriately chosen cost function of all pair-wise correlation matrices between canonical variates, ${\text{w}}_{i}^{⊤}{\text{C}}_{ij}{\text{w}}_{j}$ (i≠j ). In Kettenring (1971), five objective functions were proposed, including the sum of correlations (SUMCOR) and the sum of squares correlations (SSQCOR). The one-unit mCCA algorithm can be extended to find multiple canonical variates by using the iterative deflation method. In the first step, we simply solve (10) to obtain the first set of filters ${\text{w}}_{i,1}$ with i=1,…,D . In the next iterations, we turn (10) into a constrained optimization problem by imposing orthogonality of the form ${\text{w}}_{i,l}⊥\{{\text{w}}_{i,1},{\text{w}}_{i,2},\cdots ,{\text{w}}_{m,l-1}\}$ for all i≤D . The maximum number of canonical variates is restricted by $l\leq \text{min(}N_{i})$ .

##### ssmCCA

Similarly to standard CCA, the GraphNet ssCCA variant presented can be also extended to multiple datasets, leading to the so-called structured sparse multiset CCA (ssmCCA) (Dashtestani et al., 2022), created in the context of EEG–fNIRS fusion for group-level network connectivity analysis. The algorithm is defined as the natural combination of ssCCA and (10). In particular, ssmCCA uses SUMCOR as the multiset objective function, leading to the one-unit algorithm

$$\begin{array}{l} \underset{{\text{w}}_{1},{\text{w}}_{2},\cdots ,{\text{w}}_{D}}{\text{max}}\;\sum_{i,j=1\;\;i\neq j}^{D}\;|w_{i}^{⊤}C_{ij}w_{j}|\text{s.t. }||w_{i}|{|}_{2}^{2}\;=1,||w_{i}|{|}_{1}\leq c_{i1},\\ \;\;\;\;\;\;\;\;\;\;\;\;w_{i}^{⊤}{\text{L}}_{i}w_{i}\leq c_{i2}\;\;i=1,\ldots ,D. \end{array}$$ (11)

Here, the cross-covariance matrices were replaced by identity matrices (Witten et al., 2009), $c_{i1},c_{i2}$ are a set of hyperparameters, and ${\text{L}}_{i}$ the Laplacian matrix of dataset $X_{i}$. The extension to multiple filters can be obtained following the same logic as for mCCA (Dashtestani et al., 2022).

#### 3.6.4 Multimodal source power co-modulation (mSPoC)

EEG bandpower is proven to modulate with cognitive tasks, as well as with hemodynamic responses via the NVC, and so it is important to have data-driven fusion methods that capture such a comodulation correctly (Nunez & Srinivasan, 2006; Parra et al., 2005). Conventional methods, such as CCA, however, present some limitations to integrate bandpower signals (Dähne et al., 2013). More precisely, computing bandpower at channel level and then applying a backward linear method (5) are not in line with the assumed linear generative model of EEG (4), which harms the neurophysiological interpretation of the reconstructed sources. mSPoC (Dähne, 2015; Dähne et al., 2013) arises as a method that avoids these pitfalls by inverting the generative model prior to computing bandpower.

The method assumes that both datasets are mean free and temporally aligned with different sample dimensions $T_{x},T_{y}$. EEG recordings, x(t) , must be band-pass filtered and divided into non-overlapping time windows, indexed by e, and the fNIRS time series y(t) should be subsampled so that there is exactly one data point y(e) per EEG window. With this resampling, we can assume EEG is approximately stationary within time windows, allowing us to compute summary statistics such as average bandpower. Assuming the backward model of (5), the algorithm aims to uncover components ${\hat{s}}_{x},{\hat{s}}_{y}$, such that the temporally embedded bandpower dynamics of ${\hat{s}}_{x}$ co-modulates with ${\hat{s}}_{y}$. To this end, the source bandpower within each time window is approximated by

$$\Phi ({\hat{s}}_{x})(e)\equiv \text{Var}\left({\hat{s}}_{x}\left(t\right)\right)\left(e\right)={\text{w}}_{x}^{⊤}{\text{C}}_{x}\left(e\right){\text{w}}_{x},$$ (12)

with ${\text{C}}_{x}(e)$ denoting the within-window covariance matrix, and time delays between modalities are captured via a finite impulse response filter

$$\begin{array}{l} h(\Phi )(e)\equiv \sum_{i=1}^{N_{\tau }}w_{\tau i}\Phi (e-\tau_{i})\\ \;\;\;\;\;\;\;\;\;\;\;\;\;\;\;\;\;\;\;={\text{w}}_{x}^{⊤}\text{h}({\text{C}}_{x})(e){\text{w}}_{x},\;\;\text{h}({\text{C}}_{x})(e)\equiv \sum_{i=1}^{N_{\tau }}w_{\tau i}{\text{C}}_{x}(e-\tau_{i}), \end{array}$$ (13)

parameterized by an $N_{\tau }$-dimensional time filter ${\text{w}}_{\tau }$, and a set of pre-chosen time lags $\tau_{i}$. mSPoC is then defined by the following constrained optimization problem

$$\begin{array}{l} \underset{{\text{w}}_{x},{\text{w}}_{y},{\text{w}}_{\tau }}{\text{max}}\text{Cov}(h\left(\Phi \right)\left(e\right),{\hat{s}}_{y})\;\;\text{s.t. }\\ {\text{w}}_{x}^{⊤}{\text{C}}_{x}{\text{w}}_{x}=\;\;{\text{w}}_{y}^{⊤}{\text{C}}_{y}{\text{w}}_{y}={\text{w}}_{\tau }^{⊤}{\text{w}}_{\tau }=1. \end{array}$$ (14)

Via the method of Lagrange multipliers, (14) can be turned into a set of coupled equations that must be solved iteratively, until some convergence criterion is fulfilled. The extension to K set of filters ${\text{W}}_{x},{\text{W}}_{y},{\text{W}}_{\tau }$ can be obtained via a deflation scheme (Dähne, 2015).

#### 3.6.5 Joint ICA

Joint ICA (jICA) (V. Calhoun, Adali, Pearlson, & Kiehl, 2006) is a very straightforward method that was created in the context of unimodal multi-task fMRI analysis (V. D. Calhoun et al., 2006) and rapidly adapted to the fusion of multiple modalities such as fMRI-sMRI (V. Calhoun, Adali, Giuliani, et al., 2006), fMRI-EEG (V. Calhoun, Adali, Pearlson, & Kiehl, 2006), and fMRI-sMRI-EEG (V. Calhoun, Adali, & Liu, 2006). The method is typically used for group-level analysis, and so we now denote $N_{s},N_{x},N_{y}$ as the number of subjects, EEG and fNIRS features, respectively. Single-modality datasets, ${\text{D}}_{x}\in {\mathbb{R}}^{N_{s}\times \;N_{x}}$ and ${\text{D}}_{y}\in {\mathbb{R}}^{N_{s}\times \;N_{y}}$, are concatenated along the subject axis to generate a multimodal feature matrix $\text{D}=({\text{D}}_{x},{\text{D}}_{y})$ . jICA then assumes the following generative linear model

$$\text{D}=\text{G}{\text{V}}^{⊤}=\sum_{i=1}^{K}{\text{g}}_{i}{\text{v}}_{i}^{⊤},\;\;\text{G}\in {\mathbb{R}}^{N_{s}\times \;K},\;\;\text{V}\in {\mathbb{R}}^{\left(N_{x}+\;N_{y}\right)\;\times \;K},$$ (15)

where $K\leq \text{min}(N_{s},N_{x}+N_{y})$ is the number of components. Each of these components is characterized by an $\left(N_{x}+N_{y}\right)$ -dimensional profile ${\text{v}}_{i}$ and an $N_{s}$-dimensional vector ${\text{g}}_{i}$ that encodes the strength at which the profile is present on each subject. A standard ICA algorithm is then run on (15), assuming statistical independence between $v_{i}$, to obtain the reconstructed profiles ${\hat{\text{v}}}_{i}=({\hat{\text{v}}}_{xi},{\hat{\text{v}}}_{yi})$ , which are decomposed into modality-specific sources. Via this approach, jICA finds latent factors that commodulate between modalities and across subjects.

jICA assumes different modulation vectors ${\text{g}}_{i}$ for each subject, but the same for both modalities, giving the algorithm small flexibility. This assumption is relaxed in parallel ICA (paraICA) (J. Liu & Calhoun, 2007), another ICA extension that makes emphasis on subject-specific multimodal components. However, to the best of our knowledge, paraICA has not been used for fNIRS–EEG fusion yet.

#### 3.6.6 Survey results

We found no data-driven symmetric methods for DOT–EEG fusion. From the fNIRS–EEG survey, we extracted 14 data selection algorithms (11.2%), and 17 feature extraction ones (13.6%), most of them source decomposition (Table 4).

##### CCA in stress and workload evaluation

The advantages of implementing standard CCA for EEG–fNIRS fusion during mental stress and cognitive workload assessment were demonstrated in Al-Shargie et al. (2017b) and Ghasimi and Shamekhi (2024), respectively. Al-Shargie and colleagues used CCA analysis to reveal that the pair of canonical variates with the highest correlation (0.95) between EEG alpha bandpower suppression and decreased HbO was consistently observed in the right ventrolateral prefrontal cortex (VLPFC), indicating a highly localized area for stress-induced neural changes. This fusion approach significantly enhanced the detection of mental stress, achieving the best classification accuracy (control vs. stress), compared with using either modality alone. Moreover, CCA’s performance was also superior to the one achieved previously with jICA in Al-Shargie et al. (2016), using the same montage and mental arithmetic task. In Ghasimi and Shamekhi (2024), the authors explored the combination of six distinct fusion strategies with several classifiers for cognitive workload assessment. Among them, the combination of CCA with a linear SVM classifier achieved the highest performance for differentiating between various n-back task levels.

##### CCA and kCCA for emotion recognition

Nia et al. (2025) conducted a comparative study between concatenation, decision-level, CCA, and kCCA fusion strategies to examine the effectiveness of each framework as a hybrid fNIRS–EEG emotion recognition system. The study employs a personalized dataset of an auditory emotion-elicitation paradigm using a sparse multimodal montage over the prefrontal cortex. Power spectral density and differential entropy were used as features for both modalities. The study indicates that concatenation and decision-level fusion were both in par and outperformed the CCA-based strategies across different binary emotion classification tasks, and, on some occasions, the CCA methods performed even worse than single-modality classifiers. Such a poor performance can be attributed to a lack of auxiliary signals and physiological and motion-related noise removal for both modalities. These modality-specific confounders can not only screen shared components, harming CCA performance, but also inflate classification performance in late-fusion methods, such as concatenation and decision level, by allowing them to leverage extracerebral information. Additionally, the absence of robust regularization mechanisms in the CCA methods exacerbates overfitting issues, especially problematic given the high-dimensional input space obtained upon combining the spectral and nonlinear features.

##### CCA for motion artifact correction

Wavelet Packet Decomposition (WPD) (Mallat, 1999) was combined with CCA (WPD-CCA) in Hossain et al. (2022) for motion artifact correction, decomposing single-channel recordings into multi-channel sub-band signals. These individually generated fNIRS and EEG signals are then used as CCA inputs. The efficacy of the framework is tested on a benchmark dataset using the difference in SNR and percentage reduction in motion artifacts as metrics, concluding that WPD-CCA performs better than single-modality WPD alone.

##### tCCA in clinical contexts

In Rezaee et al. (2021), tCCA was used to detect continuous transcranial Direct Current Stimulation (ctDCS)-induced cerebral activation changes in chronic stroke survivors. There, resting-state fNIRS–EEG data from left/right prefrontal and sensorimotor cortices were collected before and after ctDCS. tCCA was applied to capture the coupling between the log10-transformed EEG bandpower (1–45 Hz) and total hemoglobin signals (HbO + HbR). Notably, significant canonical correlations were observed only in the vasomotion band (0.07–0.13 Hz), indicating that this frequency window robustly reflects the intermodal relationship, while correlations in the neurovascular coupling band (0.01–0.07 Hz) were not significant. An interesting hybrid data-driven/model-based approach was implemented in Walia et al. (2022) for exploring error-related brain states during complex surgical motor tasks. There, the authors utilized tCCA between HbO data from LS channels and EEG bandpower to extract 15 canonical EEG components. These components were used as hemodynamic regressors (replacing the typical HRF *and* boxcar function) of a GLM model, which also incorporated SS channels as confounding terms. This fusion technique demonstrated that expert surgeons exhibited a focused pattern of hemodynamic suppression in key cognitive and error-monitoring regions, while novices showed a more widespread activation. Another variation of CCA called temporally driven CCA (TdCCA) was proposed in Ran et al. (2025), where the imaginary part of coherence of the EEG signal and the fNIRS correlation coefficients in the resting and working memory state were used as inputs of standard CCA. This novel method achieved the best performance in brain connectivity classification between patients with Moyamoya disease (MMD) and control groups, compared with other methods, including sCCA and kCCA. This superior performance is attributed to TdCCA’s ability to effectively handle the temporal delays inherent in EEG–fNIRS data via the custom-tailored brain connectivity features.

##### ssmCCA for action–observation network analysis

Dashtestani et al. (2022) developed ssmCCA (11) to study multimodal, multi-participant fNIRS–EEG data fusion to characterize the human action–observation network during a live-action–observation and execution paradigm. The results from right-handed participants revealed that the left inferior parietal region was significantly activated during both paradigms. ssmCCA offered more refined spatial insights than unimodal analyses alone. Moreover, the authors compared the performance of ssmCCA with sparse mCCA and mCCA, finding that ssmCCA showed the largest correlation magnitudes in both conditions, as a consequence of being the only method among the three that handles multi-collinearity, high dimensionality, and small-sample size at the same time. The same method was later applied for NVC modeling during motor execution, observation, and imagery tasks in Su et al. (2023), validating and extending previous results.

##### mSPoC

The mSPoC algorithm was developed in Dähne et al. (2013) with the purpose of incorporating a source-decomposition method with a biologically accurate non-linear relation between EEG time courses and hemodynamic responses (as measured by fNIRS or fMRI) via EEG bandpower. The validity of mSPoC was demonstrated for simulated data and in a real-world motor execution paradigm, showing a higher performance than standard CCA in both datasets. mSPoC was used later in Ieong et al. (2019) to investigate NVC on opiate adiction, using functional connectivity-related features as model inputs. The framework allowed the finding of desynchronized alpha rhythms and reduced functional connectivity in abstinent heroin-dependent patients, indicating cerebrovascular injury in the PFC and suggesting a chronic effect from opiate exposure.

##### Other symmetric data-driven methods

During the survey, we found other novel bi-directional data-driven methods that followed different strategies from the typical source-decomposition framework. For instance, in the realm of NVC investigation, McLinden et al. (2024) and Zhu et al. (2024) developed their own fusion approaches. In the former study, instantaneous EEG power amplitude and fNIRS phase were fed into an integrated, non-linear method called electrovascular phase–amplitude coupling (PAC) with the aim of quantifying NVC during an auditory task. The Collaborative Multi-output Variational Gaussian Process Convergent Cross-Mapping (CMVGP-CCM) method was presented in Zhu et al. (2024) and used for causal analysis of EEG–fNIRS signals during an n-back paradigm, demonstrating differences in NVC under varying cognitive loads.

## 4 Methods Comparison on Simulated Data

### 4.1 Methods comparison

From our survey, we found that source-decomposition techniques were underrepresented in the literature. We, therefore, sought to evaluate them directly, as we hypothesize they are particularly well suited to continuous naturalistic brain imaging, due to their promising capabilities of disentangling the complex interplay between neural and systemic physiological activity in an unsupervised fashion, that is, without the need for labeled stimuli or events. To this end, a semi-synthetic dataset was used to compare the performance of previously reviewed methods (Section 3.6) on source reconstruction and spatial pattern estimation (Fig. 10). The dedicated goals here were to explore the differences between methods and their trends on the same benchmark, highlighting the critical roles of regularization techniques and temporal embedding. However, it is important to note that an exhaustive comparative evaluation falls beyond the scope of this review and is, therefore, reserved for future studies.

**Fig. 9.**
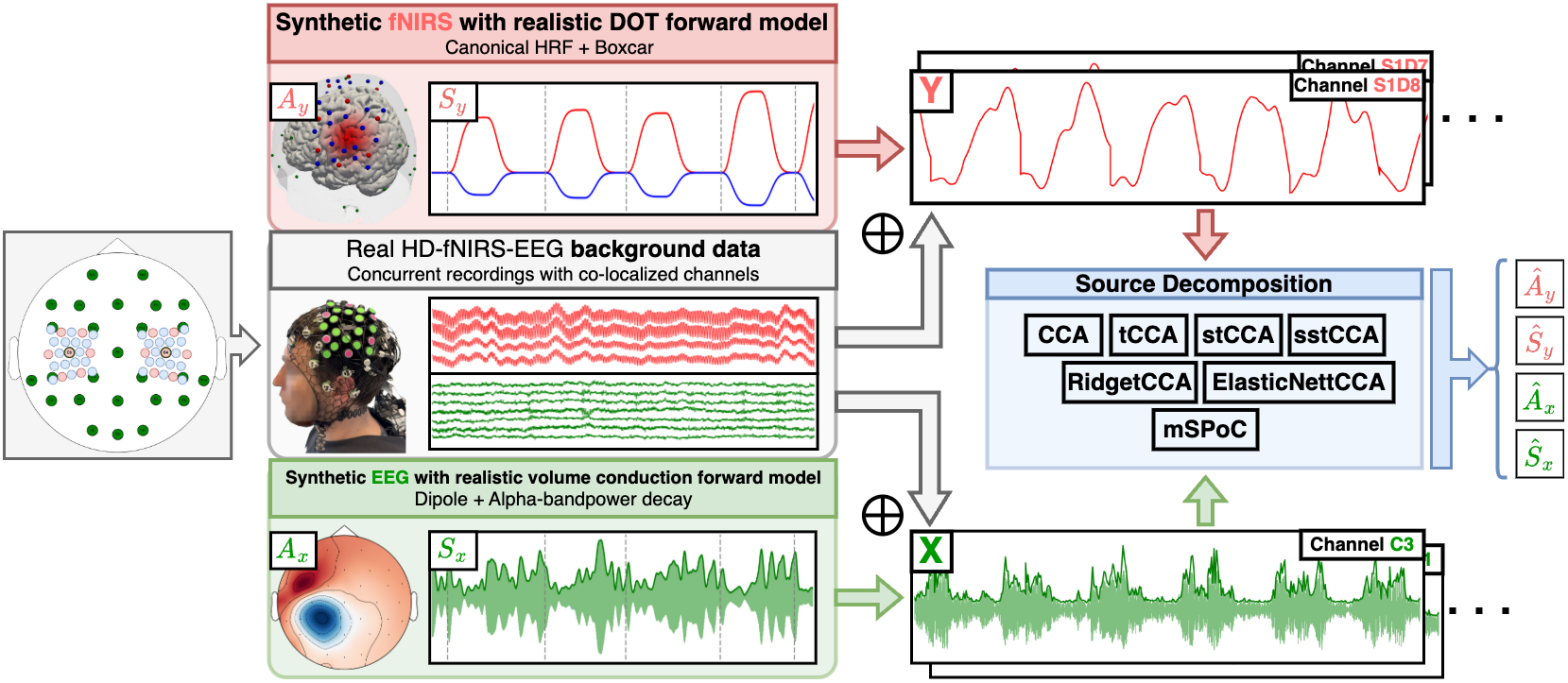
Schematics of the synthetic data pipeline and its integration into the source-decomposition methods comparison. The montage on the left shows the location of EEG channels (green), and fNIRS sources (red) and detectors (blue). The exaggerated bandpower decay in X and HbO increase in Y result from choosing a very high SNR, just for visualization purposes.

**Fig. 10.**
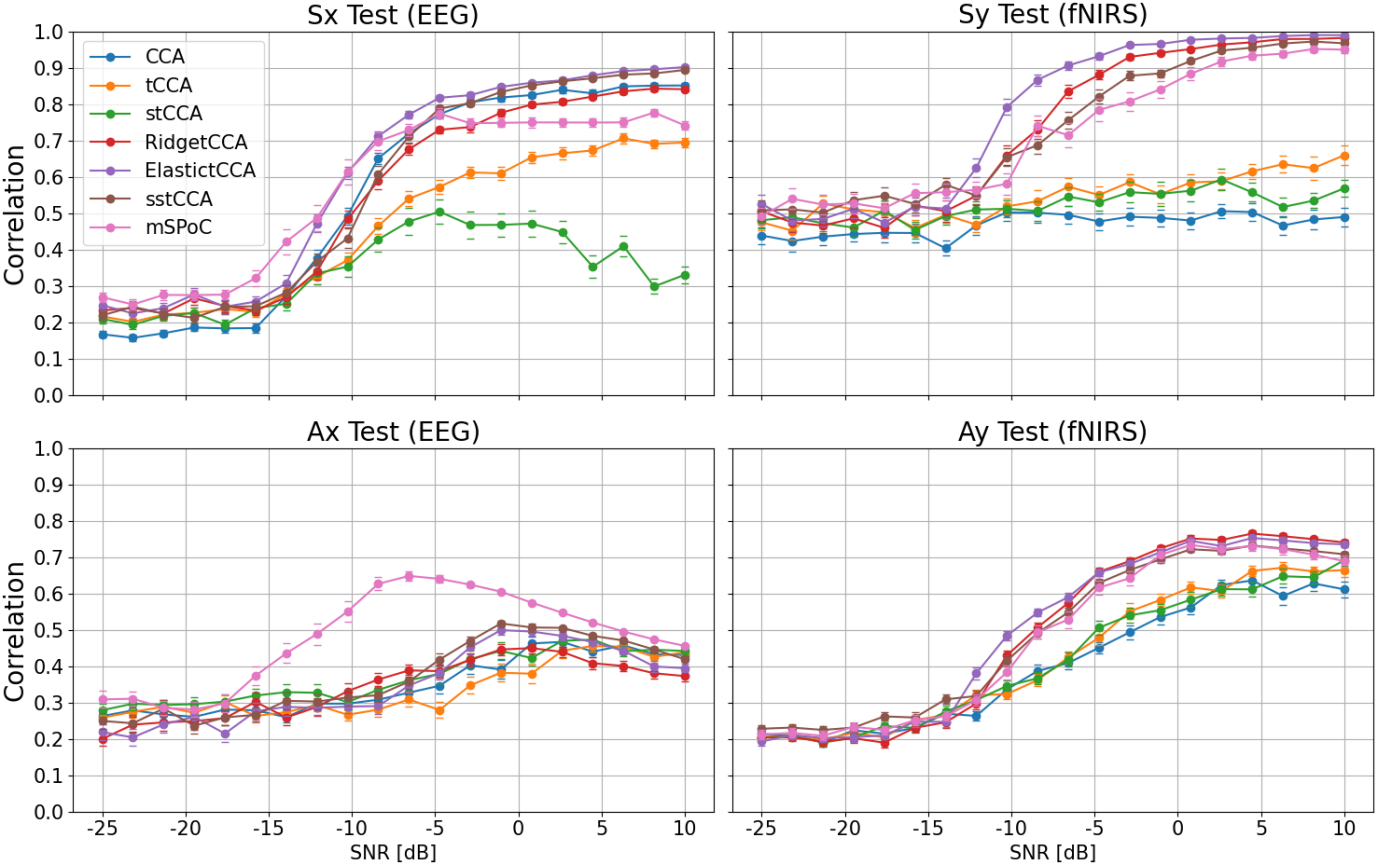
Comparison of methods’ performance for different SNR values, measured by the correlation between true and estimated quantities on a test (unseen) dataset. Each bullet point represents the bias-corrected average of such correlations across 50 simulations, and horizontal bars correspond to the average error. The first row shows correlations between reconstructed and ground-truth sources for EEG (left) and fNIRS (right), while the second row shows correlations between estimated and true spatial patterns for EEG (left) and fNIRS (right).

### 4.2 Simulated data

To systematically evaluate the performance of classical machine-learning fusion methods for EEG–fNIRS integration, it is essential to benchmark them, ideally on data with a known ground truth. However, this presents a significant challenge: publicly available EEG–fNIRS datasets are limited in number (Buccino et al., 2016; Ortega et al., 2020; Shin, Von Lühmann, et al., 2018; Shin et al., 2017), and to the best of our knowledge, there are no existing datasets providing synthetic ground-truth information in both modalities while also maintaining a realistic (physiological) noise background. This lack of accessible, well-characterized data hinders the development and validation of source-decomposition methods. To address this gap, we generated a realistic multimodal HD-fNIRS–EEG dataset with synthetic ground truth that simulates a typical motor task, such as finger tapping (Fig. 9). The resulting dataset comprises biologically plausible EEG–fNIRS neural activity, ensuring shared spatiotemporal co-modulation between sources, localized in the primary motor cortex, via simultaneous EEG alpha-band suppression and oxygenated hemoglobin increase in fNIRS. The simulated neural activity sits on top of a real concurrent HD-fNIRS–EEG recording from a single subject at rest, providing correlation between underlying processes already at noise level. By incorporating a high-density fNIRS configuration, our dataset offers an enriched platform for developing and testing fusion methods, benefiting from improved spatial resolution and enabling applications such as 3D image reconstruction through DOT. It should be noted, however, that this is not intended as the most advanced or comprehensive simulation framework; rather, it serves as a benchmarking resource to illustrate and evaluate multimodal fusion approaches within the scope of this review, with limitations and future directions discussed in Section 5. We nevertheless still describe it as “realistic” to distinguish it from fully synthetic toy datasets lacking physiological grounding, which often yield overly optimistic performance estimates.

### 4.3 Realistic HD-fNIRS–EEG data with synthetic ground truth

#### 4.3.1 Paradigm, montage, and background data

The EEG–fNIRS data simulate concurrent channel-space recordings on a single subject performing a motor task, such as finger tapping. The fNIRS dataset comprises optical-density (OD) recordings at 2 wavelengths (760 and 850 nm) across 100 measurement channels, created by pairings of 14 light sources and 32 detectors arranged in a bilateral high-density hexagonal grid over the motor cortex, with source–detector separations of 19 and 33 mm (Y. Gao, Rogers, et al., 2023). EEG data represent electrical activity collected from the 32-channel actiCAP arrangement (Brain Products GmbH, Gilching, Germany). The simulated experiment consists of one single block composed of 12 trials, each of which begins with a 10-second stimulus period followed by a recovery period whose duration is randomly chosen between 8 and 16 seconds. To produce realistic background noise underlying the simulated experiment, we collected 5 minutes of real concurrent HD-fNIRS–EEG resting-state data from a single subject, using the aforementioned montage on a custom-designed NinjaCap (von Lühmann et al., 2024). fNIRS data were recorded by two NIRSport2 devices (NIRx GmbH, Berlin, Germany) at 12.6 Hz sampling rate. EEG data were collected using a 32-channel LiveAmp amplifier (Brain Products GmbH, Gilching, Germany) with active Ag/AgCl electrodes mounted in the NinjaCap following the actiCAP arrangement, using common average reference. The sites normally occupied by FT9, FT10, T7, and T8 were repurposed to record horizontal and vertical EOG channels. Impedances were brought below 10 kΩ before data acquisition, and data were digitized at 1000 Hz. Given the importance of spatial accuracy around motor areas, two in-house co-localized fNIRS–EEG probes (Rogers et al., 2025) were placed on the C3 and C4 landmarks. As a preprocessing step on the background data, prior to synthetic data augmentation, we removed as much alpha motor activity from EEG data as possible so true sources did not interfere with our synthetically generated ones. To this end, we used the spatiospectral decomposition (SSD) algorithm (Nikulin et al., 2011) to identify alpha-band neural oscillations and removed the components whose highest activity lay in the left-motor region.

#### 4.3.2 Source spatiotemporal profile

We assumed a single common source between modalities. The source spatial profiles were created using EEG and fNIRS linear forward models that shared the same head model of the anatomy of Colin27 (Holmes et al., 1998), segmentation masks (gray/white matter, air, CSF, bone, and skin surfaces calculated using the segmentation pipeline of Harmening & Miklody (2022) based on SPM12 (Wellcome Centre for Human Neuroimaging, UCL, 2014)), FEM volume mesh (using CGAL (The CGAL Project, 2024)), and source location. The latter was chosen to be the point in the brain surface closest to the C3 landmark on the scalp. The sensitivity matrix was calculated via photon simulation using NIRFASTer (Dehghani et al., 2009) within Cedalion (IBS-Lab & Cedalion-Developers, 2024), and the EEG leadfield was computed with the SimBio FEM solver (Vorwerk et al., 2018) as integrated in the FieldTrip toolbox (Oostenveld et al., 2011). These forward matrices were then used to create fNIRS and EEG mixing matrices by combining them with modality-specific spatial information. For EEG, we used a single dipole structure and for fNIRS we generated a Gaussian-distributed activity mask, both centered around the common source location as explained above.

Simulated source time courses followed the stimulus/recovery structure of the experiment described above, where activity periods resembles biologically plausible motor activity: increasing fNIRS HbO activity while decreasing power in the alpha band (ERD). The EEG source was created using band-limited (8–12 Hz) noise modulated by slow task-related temporal envelopes, while the fNIRS HbO source was modeled using canonical hemodynamic response functions time locked to the beginning of the ERD (EEG source), including the typical hemodynamic time delay relative to activity onset. Such a synthetic hemodynamic response (together with the Gaussian-distributed spatial pattern) was generated from Cedalion’s functionalities, corresponding to the image-space extension of the method developed in von Lühmann, Li, Gilmore, et al. (2020). In each trial, the HRF amplitude was chosen randomly and proportionally to the corresponding EEG bandpower decrease. In this way, EEG source power reduction correlates with an increase in the hemodynamic response. After projecting the sources to channel space via the corresponding mixing matrices, the synthetic activity was normalized, and added to the background data, together with a scaling parameter γ that allows regulating the SNR. The latter were chosen to span from -25 dB to 10 dB, covering and extending ranges typically observed for EEG and fNIRS, and consistent with a prior multimodal simulation framework (Dähne et al., 2013).

### 4.4 Evaluation of source-decomposition methods

#### 4.4.1 Methods

We used our synthetic data to compare some of the source-decomposition methods encountered in the survey (Section 3.6): the mSPoC algorithm and temporally embedded versions of CCA, sCCA, Ridge CCA, ElasticNet CCA, and ssCCA. The regularization parameters were chosen through a coarse-grained guided search, by exploring the performance of a few runs, rather than via an exhaustive grid or random search. More precisely, we selected regularization strengths that lie midway between very low and very high penalties, yielding reasonable performance across all methods without favoring one approach over another. For sCCA we chose the L1-penalty parameters to be $\lambda_{x1}=\lambda_{y1}=0.2$ , for Ridge CCA, we chose $\lambda_{x2}=\lambda_{y2}=0.8$ as the L2-penalty parameters, and for ElasticNet CCA we used their combination.^7^ ssCCA used the same regularization parameters, while the EEG and fNIRS Laplacian matrices were built using a binary nearest neighbors approach in which the adjacency matrices contain unit values where features (channels) were closer than the (pre-defined) distances of 25 mm for fNIRS and 60 mm for EEG. Due to the time delay of the hemodynamic response, we found temporal embedding to be mandatory in all CCA-based methods to achieve satisfactory performance levels. For all of them, we chose the same set of time lags: ranging from 1 to 4 s in 1-s steps. To demonstrate the need for temporal embedding, we also tested standard CCA. mSPoC’s original formulation (Dähne, 2015) does not include any regularization mechanism (14), so we included an L2 penalty term to ${\text{w}}_{x}$ ($\lambda_{x2}=0.8$ ) together with PCA-based dimensional reduction, keeping only 0.99 of the explained variance, to obtain a competitive performance.

#### 4.4.2 Simulation and preprocessing

We ran 50 simulations with 12 trials each. Each simulation produced different temporal profiles for the EEG and fNIRS true sources due to the randomness in stimulus amplitudes and recovery times, but the source spatial profiles and the background data were always the same. For each simulation, we combined synthetic and background data using 20 different SNR values, from -25 to 10 dB. For each run and SNR, standard preprocessing pipelines were applied using Cedalion for fNIRS and the MNE toolbox (Gramfort et al., 2014; Larson et al., 2024) for EEG. fNIRS preprocessing involved channel pruning using the scalp coupling index and peak spectral power quality metrics (Pollonini et al., 2016), bandpass filtering from 0.01 to 0.6 Hz, linear detrending, and 2 Hz dowsampling. For EEG, we used linear interpolation to replace bad channels, a linear EOG regression algorithm for ocular artifact removal, and bandpass filtering in the alpha band. Then, EEG data were duplicated, and one of the copies was used to estimate its bandpower time course by splitting the data into equal-length segments and calculating the per-segment variance, resulting in an effective 2 Hz downsampling. The three datasets, namely fNIRS, EEG, and EEG bandpower time series, were segmented into individual trials, composed of the stimulus period (10 s) plus pre- and post-stimulus resting periods of constant length (8 s) each. We then performed a random 80%/20% train/test set split, resulting in 10 and 2 trials, respectively. Trials in the train set were concatenated along the time direction to generate a single time-course input dataset for each modality, while test trials were kept separated.

#### 4.4.3 Training and testing

Pairs of fNIRS and EEG sets were used as inputs of the mSPoC algorithm, while pairs of fNIRS and EEG bandpower sets entered the CCA-based methods. Each method learned a single pair of filters ${\text{w}}_{x},{\text{w}}_{y}$ from the train sets. These filters were then applied to each test trial to obtain reconstructed sources ${\hat{s}}_{x},{\hat{s}}_{y}$ via (5) and estimate spatial patterns ${\hat{\text{a}}}_{x},{\hat{\text{a}}}_{y}$ via (6). We computed the correlation between these reconstructed sources and filters and their corresponding ground-truth counterparts $s_{x},s_{y},{\text{a}}_{x},{\text{a}}_{y}$, and averaged across test trials. Figure 10 shows the bias-corrected average of such correlations for each SNR across 50 simulations, computed by first applying Fisher’s z-transformation to the correlations, averaging in z-space, and then inverse-transforming back to the original correlation space (Corey et al., 1998).

#### 4.4.4 Results

The poor performance of standard CCA on the fNIRS side ($s_{y},{\text{a}}_{y}$) demonstrates the need for temporal embedding in EEG–fNIRS fusion when using time-domain inputs. tCCA shows a slight improvement in this regard, at expenses of a trade off in the EEG side, which speaks in favor of incorporating some regularization mechanism. This is confirmed by the overall best performance achieved by ElasticNet tCCA on three of the reconstruction tasks. For EEG spatial pattern estimation, however, mSPoC achieves the best overall correlation, which is not surprising considering it was introduced exactly for the purpose of dealing with bandpower EEG data in a way consistent with the linear forward model (4). sstCCA comes second in ${\text{a}}_{x}$ estimation due to the use of local spatial information, although it performs slightly worse that ElasticNet in the rest of the tasks.

It is worth highlighting that such an assessment of the reviewed source-decomposition methods is far from exhaustive: it represents only a small proof-of-concept comparison and does not sweep across all possible algorithms, parameter settings, or regularization strategies. In particular, we did not study the temporal patterns learned by mSPoC, encoded in ${\text{w}}_{\tau }$, that theoretically should resemble the ground-truth HRF from the fNIRS simulated data (Dähne, 2015). This is a key ingredient, specific to mSPoC, that gives the method an advantage over the others. We leave a more comprehensive evaluation and analysis of these and other topics for future work. To foster transparency and make it easy for others to build on this study, we provide full implementations of each method in Cedalion^8^ together with a Jupyter notebook that reproduces these results.^9^ We encourage researchers to extend this work by conducting similar systematic comparisons on additional benchmarks.

## 5 Current Limitations and Future Directions

### 5.1 Current challenges

Despite the growing interest and progress in multimodal EEG–fNIRS/DOT fusion for continuous brain imaging, several key limitations still hinder the field’s full potential. First and foremost is the unequal treatment of the two modalities in most studies. EEG data typically benefit from extensive artifact removal techniques and auxiliary recordings (e.g., EOG, EMG), while fNIRS preprocessing often remains limited to motion correction and basic filtering. This imbalance results in suboptimal fusion, where noise and systemic physiology in fNIRS are insufficiently addressed, ultimately degrading the quality of the integrated signal (Scholkmann et al., 2022). From a methodological perspective, non-directional and late-stage fusion strategies dominate the literature, while unsupervised, data-driven symmetric methods, particularly source-decomposition approaches, remain underutilized. While this may not represent a problem in conventional neuroscientific scenarios, it poses both a challenge and opportunity if addressed for continuous and naturalistic brain imaging. Most of the algorithms used in many of the reviewed articles may currently be limited in their ability to uncover rich cross-modal relationships. In this context, source-decomposition methods promise alternatives for greater flexibility, interpretability, and better real-time, single-trial analysis. However, another major challenge lies in the limited availability of publicly accessible fNIRS–EEG datasets and the complete absence of high-density or synthetic extensions. This lack of resources significantly hinders objective benchmarking of fusion methods and undermines reproducibility across studies. While our realistic synthetic dataset represents an initial step toward addressing this issue, there remains a critical need for more diverse and ecologically valid datasets that incorporate controlled artifacts, auxiliary signals, and/or known ground-truth activation patterns.

### 5.2 Limitations

While our proof-of-concept comparison offers valuable insights, some limitations must be acknowledged. First, the analysis did not include an exhaustive hyperparameter search or systematic sensitivity analysis for each algorithm, so alternative parameter settings may yield different relative performances. We believe that such an investigation could favor the most complex methods, namely (regularized) mSPoC, ssCCA, and kCCA, since they are the ones that depend on a larger parameter space. Second, guided by the review’s main focus on naturalistic scenarios, we only compared data-driven, symmetric fusion methods and did not benchmark them against model-based, asymmetric, or directionally informed techniques, limiting the generalizability of our findings to that particular class of algorithms (see Nia et al., 2025 for a first step in this direction). Finally, although our synthetic dataset was designed to mimic key aspects of continuous EEG–fNIRS recordings, it does not capture the full complexity of real-world signals (Pfurtscheller & Lopes da Silva, 1999). In particular, our synthetic dataset does not model the transient ipsilateral motor-cortex desynchronization that often precedes or accompanies contralateral mu-band ERD in simple movement tasks (Muellbacher et al., 2000). We have also omitted the characteristic post-movement beta rebound (beta-band event-related synchronization) observed in sensorimotor cortex following movement termination (Jurkiewicz et al., 2006). Such omissions may lead to deviations from real data, which we expect to primarily increase variability in the results when comparing fusion methods, rather than systematically biasing performance in favor of one method over another. Moreover, the resting-state background activity was derived from a single subject. While this provides a controlled test bed for benchmarking methods in this manuscript, it does not reflect the inter-individual variability in physiological noise typically encountered in empirical datasets. Incorporating these phenomena would further improve the physiological realism of our simulations and provide a more rigorous testbed for fusion algorithms.

### 5.3 Future directions

Looking forward, we identify several important directions for future work. First, greater integration of physiological priors and auxiliary measurements, such as heart rate, respiration, and SS channels, will be crucial for improving artifact rejection and isolating neural signals (Scholkmann et al., 2022; Yücel et al., 2021). A second promising direction lies in the development of more sophisticated classical machine-learning fusion methods that integrate spatial and/or context-aware priors. In particular, we identify mSPoC-like approaches, which already incorporate more accurate physiological models, as strong candidates for such enhancements. These methods could be significantly improved by embedding regularization techniques, including not only traditional L1/L2 penalties, but also structured spatial priors derived from anatomical layouts or sensitivity profiles, such as those used in fNIRS/DOT-informed fusion (Cao et al., 2021). Furthermore, these methods could also be enhanced by data-driven constraints, derived from auxiliary recordings, a technique that has been successfully implemented already in the context of single-modality (Lu & Rajapakse, 2000; Yang et al., 2023) and multimodal (J. Liu & Calhoun, 2007) decomposition, although never in fNIRS–EEG fusion.

As a third future direction, it would be highly beneficial to apply these advanced source-decomposition frameworks and the next generation of algorithms built upon them to scientific questions that our survey did not address. The infra-slow EEG–fNIRS coupling is one of these examples (Pfurtscheller et al., 2012; Watson, 2018), in which one could look for shared sources underlying <0.1 Hz EEG DC shifts and fNIRS hemodynamic oscillations, to test hypotheses about slow neurovascular autoregulation, and resting-state network dynamics (Thompson et al., 2014). Another compelling direction would be the generalization of these source-decomposition methods for the fusion of other modalities, such as fMRI-EEG, exploiting the similarities between (fast) fMRI and fNIRS/DOT in volume space. Moreover, such methods could be further extended for tri-modal fusion, such as fMRI–fNIRS–EEG (Anwar et al., 2016; Muthalib et al., 2013). While not directly applicable to naturalistic scenarios, (fast) fMRI could complement fNIRS rapid hemodynamic acquisition and enhance fusion by its unique full-volume spatial resolution. For such extension, one could build on top of the tools already developed in V. Calhoun, Adali, and Liu (2006), a successful case of tri-modal integration covered in the review, which demonstrated the feasibility of feature-based fusion across functional MRI, structural MRI, and EEG.

Lastly, the field would benefit from community-driven open-source toolkits for multimodal data-driven analysis and datasets. This is one of the main motivators for our development of the Cedalion toolbox (IBS-Lab & Cedalion-Developers, 2024): With Cedalion, we aim to enable state-of-the-art multimodal fNIRS methods development and benchmarking, and to foster reproducibility and standardized evaluation protocols. Regarding community-driven open-source datasets, a public challenge on fNIRS–EEG fusion would be a highly promising step toward this direction by promoting open data sharing and accelerating methodological innovation, much like the successful impact of competitions in the EEG-based BCI field (Blankertz et al., 2006; Tangermann et al., 2012). It is our intention to engage in this direction in the near future by releasing a challenge on single-trial brain activity decoding on a systemic physiology-augmented fNIRS–EEG dataset.

We believe that pursuing all of these avenues combined will advance the field of fNIRS–EEG fusion for continuous brain imaging toward more robust, interpretable, and reproducible brain–body investigations.

## Data Availability

All compared source-decomposition methods can be found in the “sigdecomp” package^10^ of our Python-based fNIRS/DOT analysis toolbox Cedalion (IBS-Lab & Cedalion-Developers, 2024). We also provide an example notebook containing the methods comparison on our synthetic data to enable the reproducibility of our results.^11^

## Footnotes

1
https://github.com/ibs-lab/cedalion/tree/dev/src/cedalion/sigdecomp/multimodal

2
https://github.com/ibs-lab/EEG-fNIRS-Fusion/tree/main/multimodal_fnirs_eeg_sensor_fusion_review

3 By low-process we mean operations whose outputs are multivariate time-series, the same data type as raw sensor data. Any derived quantity that is not of this kind here is defined as a feature.

4 Statistics are inflated due to unrelated keyword matches, leaving only 35 deduplicated articles when “DOT” is excluded.

5 Although adoption has increased in recent years with improved hardware and software support, only 10 of the 124 surveyed studies included SS channels, so they remain rarely recorded overall.

6 Unless stated otherwise, we use an abstract feature-sample notation whose interpretation depends on the level of analysis, such as single-trial or group-level. For instance, features can refer to channels or voxels, while samples can be time-points, trials, or subjects.

7 See Cedalion’s implementation for a detailed description of each parameter.

8
https://github.com/ibs-lab/cedalion/tree/dev/src/cedalion/sigdecomp/multimodal

9
https://github.com/ibs-lab/EEG-fNIRS-Fusion/tree/main/multimodal_fnirs_eeg_sensor_fusion_review

## Supplementary Materials

Example notebook containing the methods comparison on our synthetic data: https://github.com/ibs-lab/EEG-fNIRS-Fusion/tree/main/multimodal_fnirs_eeg_sensor_fusion_review.

## Appendix A

The following tables contain surveyed articles that used nondirectional fusion strategies (Fig. 2). Stats refers to statistical quantities such as mean, maximum, slope, variance, skewness, kurtosis, or median, applied to raw time-course data. PSR: Phase-Space Reconstruction, TPF: Time-Phase-Frequency features, FBCSP: Filter-Bank Common Spatial Pattern (Ang et al., 2008).

**Appendix Table A1.** Surveyed studies that used a concatenation fusion strategy.

Reference	EEG feature	fNIRS feature	Study objective
Almajidy et al. (2015)	PSD	HbO slope	BCI classification
Buccino et al. (2016)	CSP + Bandpower	HbO, HbR CSP + stats	
Banville et al. (2017)	Bandpower	HbO, HbR mean	
Ge et al. (2017)	PSR + CSP	HbO Hurst exponent	
Mandal et al. (2020)	Stats	HbO, HbR stats	
Al-Quraishi et al. (2021)	ERD	HbO, HbR stats	
Alhudhaif (2021)	Stats	HbO, HbR stats	
Z. Wang et al. (2023)	CSP	HbO, HbR CSP	
Ergün et al. (2024)	Bandpower	HbO, HbR bandpower	
Y. Sun et al. (2020)	PSD	HbO, HbR stats	
Fu et al. (2017)	TPF	HbO, HbR raw time-course	
X. Lin et al. (2018)	ERP	HbO raw time-course	
Firooz and Setarehdan (2019)	Bandpower + PSD	HbO stats	
Cao et al. (2022)	PSD and FC	HbO, HbR raw time-course	
Gamboa et al. (2022)	Stats	HbO, HbR stats	
Zakeri et al. (2023)	Bandpower	HbO, HbR raw time-course	
Gemignani and Gervain (2024)	Raw time-course	HbO, HbR raw time-course	
Guo et al. (2024)	FBCSP	HbO, HbR CSP + Relief	
Y. Meng et al. (2025)	FBCSP	HbO, HbR stats	
Abtahi et al. (2020)	PSD	HbO, HbR raw time-course	Clinical assessment
Guevara et al. (2020)	Raw time-course	HbO, HbR raw time-course	
Güven et al. (2020)	Complexity + ERP	HbO raw time-course	
Liang et al. (2022)	ERD/ERS	HbO raw time-course	
Maddury (2024)	TPF	HbO, HbR TPF	
Y.-C. Jiang et al. (2022)	ERD/ERS	HbO raw time-course	Method presentation

**Appendix Table A2.** Surveyed studies that used a decision-level fusion strategy.

Reference	EEG feature	fNIRS feature	Study objective
Pfurtscheller (2010)	ERS/ERD	HbO raw time-course	BCI classification
Fazli et al. (2012)	FBCSP	HbO, HbR mean	
Tomita et al. (2014)	SSVEP	HbO, HbR slope	
Putze et al. (2014)	Bandpower + ERP	HbO, HbR mean	
Blokland et al. (2014)	PSD	HbO, HbR raw time-course	
Koo et al. (2015)	Bandpower	HbO slope	
Lee et al. (2015)	FBCSP	HbO, HbR mean	
Ahn et al. (2016)	PSD	HbO, HbR raw time-course	
Y. Liu et al. (2017)	PSD	HbO, HbR mean	
Nguyen et al. (2017)	PSD	HbO, HbR raw time-course	
Gupta et al. (2017)	ERS/ERD	HbO, HbR stats	
Al-Shargie et al. (2017a)	Bandpower	HbO raw time-course	
Shin, Kim, et al. (2018)	FBCSP	HbO, HbR mean + slope	
Shin, Kwon, and Im (2018)	FBCSP	HbO, HbR stats	
Shin, Müller, and Hwang (2018)	FBCSP	HbO, HbR mean + slope	
Rezazadeh et al. (2019)	DWT	THbO, HbR stats	
X. Jiang et al. (2019)	Multiple feature paths	Multiple feature paths	
Kwon et al. (2020)	FBCSP	HbO, HbR mean	
Han et al. (2020)	FBCSP	HbO, HbR mean + slope	
Qiu et al. (2022)	TPF	HbO, HbR TPF	
